# Characterizing nutrient uptake kinetics for efficient crop production during *Solanum lycopersicum var*. *cerasiforme* Alef. growth in a closed indoor hydroponic system

**DOI:** 10.1371/journal.pone.0177041

**Published:** 2017-05-09

**Authors:** Ju Yeon Lee, Arifur Rahman, Hossain Azam, Hyung Seok Kim, Man Jae Kwon

**Affiliations:** 1 Korea Institute of Science and Technology, Gangneung, Republic of Korea; 2 Korea University, Seoul, Republic of Korea; 3 Civil and Environmental Engineering, The George Washington University, Washington, DC, United States of America; 4 Civil and Environmental Engineering, Manhattan College, Riverdale, NY, United States of America; INRA, FRANCE

## Abstract

A balanced nutrient supply is essential for the healthy growth of plants in hydroponic systems. However, the commonly used electrical conductivity (EC)-based nutrient control for plant cultivation can provide amounts of nutrients that are excessive or inadequate for proper plant growth. In this study, we investigated the kinetics of major and minor nutrient uptake in a nutrient solution during the growth of tomato (*Solanum lycopersicum var*. *cerasiforme* Alef.) in a closed hydroponic system. The concentrations of major and minor ions in the nutrient solution were determined by various analytical methods including inductively coupled plasma-optical emission spectroscopy (ICP-OES), ion chromatography (IC), ion specific electrodes, and/or colorimetric methods. The concentrations of the individual nutrient ions were compared with changes in the EC. The EC of the nutrient solution varied according to the different growth stages of tomato plants. Variation in the concentrations of NO_3_^−^, SO_4_^2−^, Mg^2+^, Ca^2+^, and K^+^ was similar to the EC variation. However, in the cases of PO_4_^3−^, Na^+^, Cl^−^, dissolved Fe and Mn, Cu^2+^, and Zn^2+^, variation did not correspond with that of EC. These ions were generally depleted (to 0 mg L^−1^) during tomato growth, suggesting that these specific ions should be monitored individually and their supply increased. Nutrient uptake rates of major ions increased gradually at different growth stages until harvest (from < 3 mg L^−1^ d^−1^ to > 15 mg L^−1^ d^−1^). Saturation indices determined by MINEQL+ simulation and a mineral precipitation experiment demonstrated the potential for amorphous calcium phosphate precipitation, which may facilitate the abiotic adsorptive removal of dissolved Fe, dissolved Mn, Cu^2+^, and Zn^2+^.

## Introduction

The total area of crops cultivated using hydroponic systems has expanded rapidly worldwide [1]. In recent years, more efficient use of water and fertilizers, together with better control of climate and pests has ensured significant increases in crop production using hydroponic systems worldwide [2–3]. However, almost all soil-less hydroponic systems are open systems, with no collection and recirculation of drainage water from substrate/nutrient containers. Therefore, drainage water is typically discharged to the surrounding environment without proper treatment, contaminating the associated soil, groundwater, and water bodies. Recently, increasing demand for water in domestic, industrial, environmental, and recreational sectors has forced agriculturists to manage irrigation water carefully, contributing to environmental preservation. This demand has resulted in the shift from open hydroponic systems to closed hydroponic systems. In general, closed hydroponic systems have lower water and nutrient requirements for plant growth [1–3] due to the recycling of water and nutrients. Additionally, indoor farming system have some advantages over open field systems. Environmental conditions for crop cultivation can be better controlled in indoor farming compared to an open field system, which relies on soil, sunlight, and irrigation. An indoor farming system can be equipped with an automatic climate control system and artificial lighting rather than depending on sunlight, which is beneficial for crop production.

The most important aspect of crop production in a field, greenhouse, or hydroponic system is the availability and supply of balanced nutrients for the plants. However, a deficiency or excess of nutrients is frequently observed over long-term cultivation in closed hydroponic systems, impairing the potential growth of the plants [4].

In hydroponic systems, plants are typically nurtured with mixed nutrient solution, with a relatively high concentration of nutrients, adjusted according to its electric conductivity (EC). The EC is proportional to the total dissolved ions present in the solution, making it an effective measure of nutrient solution strength. Thus, EC has been used to estimate nutrient requirements in a recirculating nutrient solution system [3]. However, EC indicates total dissolved ion concentrations only, and cannot be used directly to determine individual ion concentrations [3]. Therefore, EC-based nutrient control for plant cultivation may provide amounts of nutrients that are excessive or inadequate for the plants. For example, tomatoes often require high amounts of calcium (Ca) and potassium (K) to produce high quality fruits [5–6]. Thus, adjustments required to supply the appropriate amount of specific ions should be decided based on the rate and extent of nutrient removal, as well as accommodation of the phases of tomato growth and fruiting.

Although plant uptake of nutrient ions in open hydroponic growth systems has frequently been investigated [2, 7–11], the dynamic behavior of major and minor nutrients in indoor closed hydroponic systems, and the causes of deficiency or excess of specific ions during tomato growth is poorly investigated. Furthermore, no major study has looked into the comparability and reproducibility of the specific ion concentrations determined by different analytical methods (conventional instrumental analysis vs. quick assay using ion specific electrodes or commercial kits). The concentration of individual nutrient ions is often measured on-site, using simple analytical procedures, because lab-based analytical services are not always available and sometimes require weeks for interpretation of results. Additionally, some nutrient ions should be supplied within a narrow time window (e.g., a few days) to ensure better plant growth. This constraint has created the demand for the development or identification of efficient, robust, and inexpensive analytical tools that can be used repeatedly in the field under realistic conditions [12].

With this aim, we monitored variation in concentration of the major and minor ions in the nutrient solution supplied for tomato growth in a closed hydroponic indoor farming system. The specific objectives of this study were 1) to investigate the dynamics of the uptake of major and minor nutrients during tomato growth in an indoor hydroponic farming system, 2) to identify deficiencies of specific ions in the nutrient solution during cultivation, and 3) to evaluate whether ion concentrations determined by rapid assay or ion-specific electrodes on-site are adequately comparable to those determined by relatively precise analytical instruments in the laboratory.

## Materials and methods

### Experimental set-up of pilot-scale indoor farming system

This study was performed at an indoor hydroponic farm operated by the Korea Institute of Science and Technology (KIST) at Gangneung, South Korea. The self-pollinating tomato cultivar (*Solanum lycopersicum var*. *cerasiforme* Alef.) was purchased from Jeil Seed Co. (South Korea) and was seeded in 96 pots. The seeds were grown in rockwool cubes [25 cm (W) × 2.5 cm (L) × 3.5 cm (H)] (Grodan Co., Denmark) with tap water in a pilot-scale indoor farming system. The indoor farming system had approximately 100 m^2^ of space comprising vertical cultivation beds, a closed nutrient solution circulating system, an automatic climate control system, and a light emitting diode (LED) lighting system (multi wavelengths 370–780nm, 12W)(MK3505, Taejong, South Korea).

**Fig 1:**
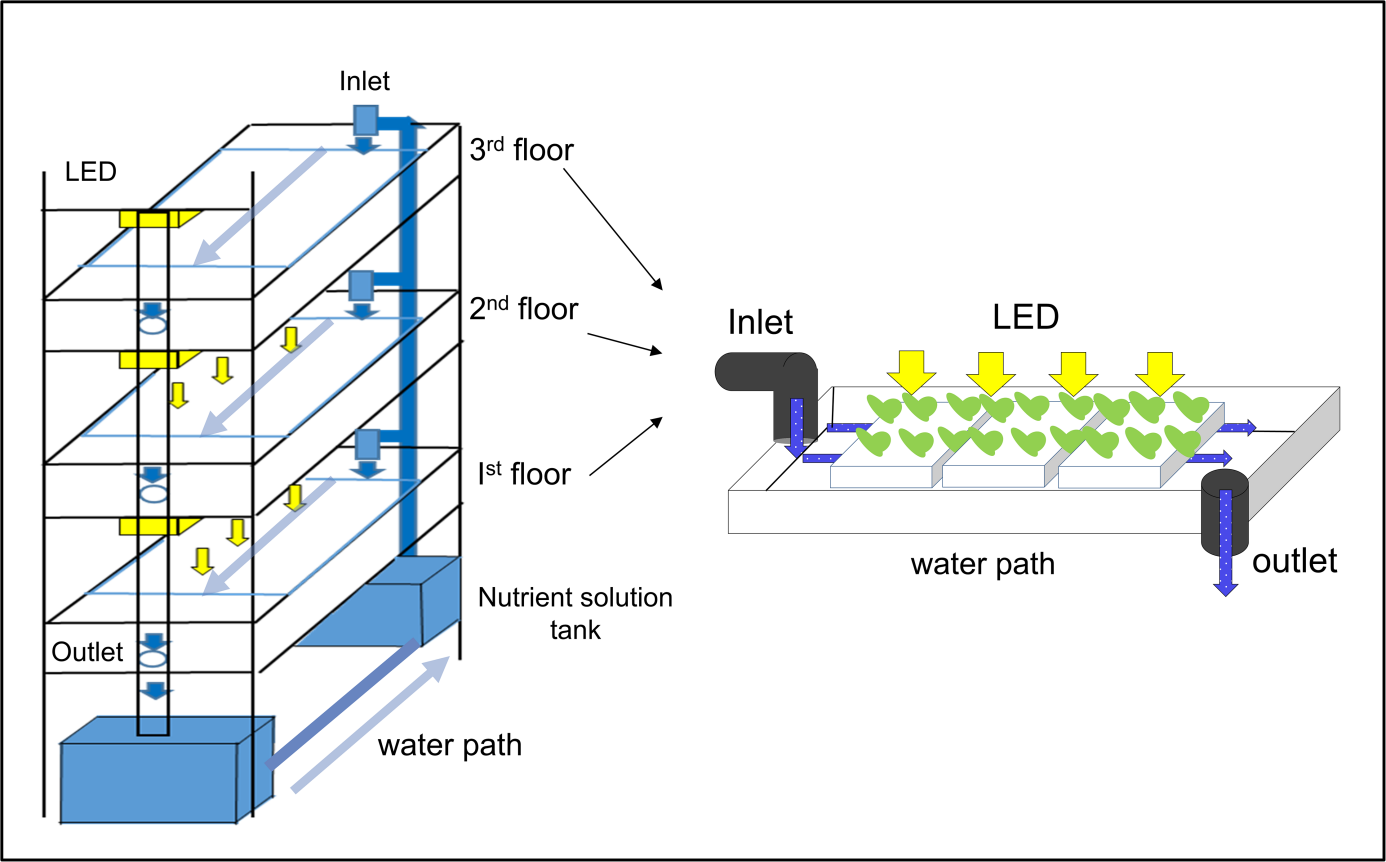
Schematic diagram of the hydroponic indoor farming system used in this study.

Three stacked floor beds were used to grow tomatoes, with each bed containing 32 tomato seedlings. A schematic diagram of the hydroponic system used in this study is shown in Fig 1. We transplanted 32 seedlings into each high-density polystyrene box [0.9 m (W) × 0.56 m (L); 72 holes] in each deep-flow culture bed [65 cm (W) × 3 m (L) × 20 cm (H)]. For proper mixing, the nutrient solution was circulated continuously at a rate of 19 L min^–1^ using an electric pump placed after the mixing tank. As the water level in the cultivation beds fell below 60% of the initial water level due to evaporation and/or evapotranspiration by tomato plants, the mixing tank was refilled with freshly prepared nutrient solution. The nutrient solution was also replenished with new nutrient solution when the target EC (approximately 1.5 mS cm^–1^) became difficult to maintain due to extreme consumption of nutrients from the solution by the plants.

Cultivation began on August 18, 2015, by transplanting 8-day-old seedlings, and lasted for approximately 115 d. The air temperature and relative humidity (RH) in the farm space were maintained at 21.7 ± 4.6°C and 61.6 ± 14.1%, respectively. Light intensity above the seedlings was approximately 200 μmol m^–2^ s^–1^ (light/dark = 15:9 h). Table 1 describes the environmental conditions during cultivation, sampling time, and nutrient solution injection time. The average, minimum, and maximum values of atmospheric temperature, relative humidity, water temperature, and dissolved oxygen (DO) concentration, light intensity, pH, and EC are reported for the pilot-scale hydroponic system. The temporal variation in atmospheric and nutrient solution conditions during tomato growth is shown in S1 Fig.

**Table 1:** Summary of cultivation condition, sampling time, and nutrient solution injection time.

Parameters	Average	Min	Max
Atmospheric temperature (°C)	21.7	17.1	24.8
Humidity (%)	61	47	76
Water temperature (°C)	23.2	18.2	27.3
Dissolved oxygen (mg L^−1^)	8.3	7.6	8.8
Light intensity (μmol m^−2^ s^−1^)	198	101	337
pH	7.6	5.8	8.3
EC (mS cm^−1^)	1.07	0.35	1.49
Sampling point (measured time) (n = 45)	Stage I: Day 0, 1, 2, 4, 6, 8, 10, 12 Stage II: Day 14, 16, 18, 20, 22, 24, 27 Stage III: Day 33, 36, 39, 42, 45, 49 Stage IV: Day 52, 56, 59, 63, 66, 69, 72, 76, 79, 83, 86, 90, 93, 97, 100, 104, 107, 111		
Nutrient solution injection time (n = 12)	Stage I: Day 0, 12 Stage II: not supplied Stage III: Day 34, 45 Stage IV: Day 54, 63, 70, 79, 84, 91, 97, 104		

Each bed was filled with approximately 60 L of nutrient solution. The concentrated nutrient solution was purchased from Gafatech (Hwaseong, South Korea) (S1 Table) and was mixed with tap water. The tap water used in this experiment contained low sodium (Na) and chlorine (Cl) concentrations (10 mg L^–1^ Na^+^ and 16 mg L^–1^ Cl^–^). The composition of diluted nutrient solution for tomato growth (in mg L^–1^) was nitrate-N (NO_3_^–^-N, 112.5), phosphate-P (PO_4_^3–^-P, 22.6), sulfate (SO_4_^2–^, 142.2), chloride (Cl^–^, 12.4), potassium ion (K^+^, 117.2), calcium ion (Ca^2+^, 109.3), magnesium ion (Mg^2+^, 30.4), sodium ion (Na^+^, 13.1), dissolved iron (Fe_diss_, 0.7), dissolve manganese (Mn_diss_, 0.2), copper ion (Cu^2+^, 0.6), and zinc ion (Zn^2+^, 0.2). The nutrient solution in this study was supplied at EC values of 1200–1490 μS cm^–1^ (Table 1). The nutrient solution was not specifically buffered. The plant cultivation was divided into four different phases/stages: Stage I (Transplanting), Stage II (Adaptation), Stage III (Flowering), and Stage IV (Fruit set). The addition of nutrient solution (or injection) at different stages based on specific ion concentration measurements is described in Table 1.

### Sampling and analytical methods

A 160-mL liquid sample was collected at the output (Fig 1), and immediately filtered through 0.45-μm nylon membrane filters (GE, Germany) to remove microorganisms and fine suspended particles. Then 10-mL aliquots of the filtrate were used to measure anions and cations. The concentrations of NO_3_^–^-N, PO_4_^3–^-P, Cl^–^, and SO_4_^2–^ were determined using single-column ion chromatography (Metrohm 850 Professional IC, Switzerland). Dissolved cations were analyzed using a Varian 730-ES inductively coupled plasma-optical emission spectrophotometer (ICP-OES, PerkinElmer SCIEX, USA) after acidification with an 1% (v/v) nitric acid. Ammonium was analyzed using an ammonium assay with ultraviolet (UV) spectrophotometer (DR2800, HACH, Loveland, CO, USA) at 670 nm [13]. Total nitrogen (T-N) and total phosphorus (T-P) in 15-mL water samples were determined by the Standard Methods 4500-N C Persulfate Method, and the 4500-P B Acid Persulfate Digestion Method, for water and wastewater, respectively [14–15]. The concentrations of Cl^–^, K^+^, and Na^+^ were measured using 40-mL water samples with ion-specific electrodes (ISE) according to manufacturer instructions: Orion VERSA STAR No. 9617BNWP (Cl^–^), No. 9719BNWP (K^+^), and No. 8611BNWP (Na^+^) (Thermo Scientific, USA). The nutrient uptake rate of cations and anions was calculated as the slope of decrease of ion concentration with corresponding time (operational day) for each spike of nutrient solution added and expressed as mg L^–1^ d^–1^.

Air temperature and relative humidity (RH) were measured using a thermometer/humidity meter (YTH-104, UINS Co., South Korea). The average height (cm) of the tomato stems was determined by measuring the stem lengths of 39 individual plants at each sampling time. The solution pH and EC were measured directly in the cultivation bed using an Orion Star A325 pH and EC meter, and the dissolved oxygen (DO) was analyzed using an Orion Star A326 DO meter (Thermo Scientific, USA).

All analytical procedures were validated using certified and/or internal reference materials. The detection limits and instrumental calibration ranges are shown in S2 Table. All chemicals used were of reagent grade quality or higher. Distilled deionized water (ddH_2_O) (> 18.2 MΩ cm) from a Millipore ultrapure water purification system (Barnstead, USA) was used throughout the study.

### Saturation index (SI) computation

The potential for mineral precipitation in the nutrient solution during tomato plant growth was computed using MINEQL+ version 4.6 software. The SI is a logarithmic value of the ratio between the ion product of the solid and the solubility constant of a particular solid, and indicates the condition of the nutrient solution, where SI > 0 indicates supersaturation (potential for mineral precipitation) and SI < 0 indicates under-saturation (precipitation unlikely).

### Statistical analysis

The Pearson correlation coefficient was employed (using SYSTAT 10.2) to determine potential relationships between EC, pH and major and minor ion concentrations with operational day of the pilot-experiment. The significance of nutrient uptake rate between major ions at transplanting, flowering and fruit-set stages was evaluated by performing two-sample t-test (unequal variances) for p-value determination (5% significance level). The similar t-test was also conducted to verify the statistical significance between measurements of specific ion (K^+^, Na^+^, Cl^–^, NO_3_^–^, PO_4_^3–^) concentrations by various analytical measurements (i.e., cations by ICP-OES and anions by IC) and on-site measurements (i.e., K^+^, Na^+^, Cl^−^by ISE and NO_3_^–^ and PO_4_^3–^ by commercial kit) evaluated in this study.

### Batch experiments to detect mineral precipitation from nutrient solution

Batch experiments were conducted using 4 × 2-L Pyrex bottles; the experiments were performed over 3 d. The bottles were filled with 2 L of nutrient solution. The nutrient solution was synthesized such that its chemical composition was similar to that used for tomato growth. The composition of the synthesized nutrient solution (in mg L^–1^) was NO_3_^–^-N (107.3), PO_4_^3–^-P (21.8), SO_4_^2–^ (64.4), Cl^−^(8.6), K^+^ (107.5), Ca^2+^ (76.0), Mg^2+^ (15.3), Na^+^ (38.1), Fe_diss_ (0.9), Mn_diss_ (0.2), Cu^2+^ (0.3), and Zn^2+^ (0.2). The target EC was 1.5 mS cm^–1^. The pH was maintained between 8.0 and 8.5 using 0.05 N NaOH. The medium was aerated by direct injection of ambient air at the rate of 1.875 mL min^–1^. The tops of the bottles were covered with sterilized cotton to inhibit evaporation. Light intensity above the solutions was approximately 200 μmol m^–2^ s^–1^.

Aqueous samples were collected at days 0 and 3. A 10-mL aqueous sample was taken from each bottle with a sterilized pipette tip and immediately filtered through a 0.45-μm nylon membrane filter (GE, Germany) to remove fine suspended particles. The filtrate was then used to measure anions and cations. The solution pH and EC were measured directly from the top of the bottle using an Orion Star A325 pH and EC meters. The sample was analyzed to determine the Ca/P ratio of the solution.

Solid samples precipitated from nutrient solution were taken at the end of the experimental period (day 3). Samples were analyzed to determine the Ca/P ratio, morphology, and mineralogy of the powder. The aqueous sample (approximately 1980 mL) was filtered through filter paper using a vacuum pump, and then the filter paper was dried. The dried powder was digested with concentrated hydrochloric acid (4 mL). The extracts were then diluted with 1% (v/v) nitric acid and analyzed by ICP-OES. The morphology of the dried powder (filtered precipitates) was determined using a scanning electron microscope (SEM; S-3000H Hitachi at KIST, Gangneung, South Korea). The mineralogy of the precipitate was analyzed using X-ray diffraction spectroscopy (XRD) with voltage and current settings of 40 kV and 30 mA, respectively, under Cu-Kα radiation (1.54060 Å) at the Korea Basic Science Institute (Seoul, South Korea).

## Results

### Phases of tomato growth using EC as an indicator

**Fig 2:**
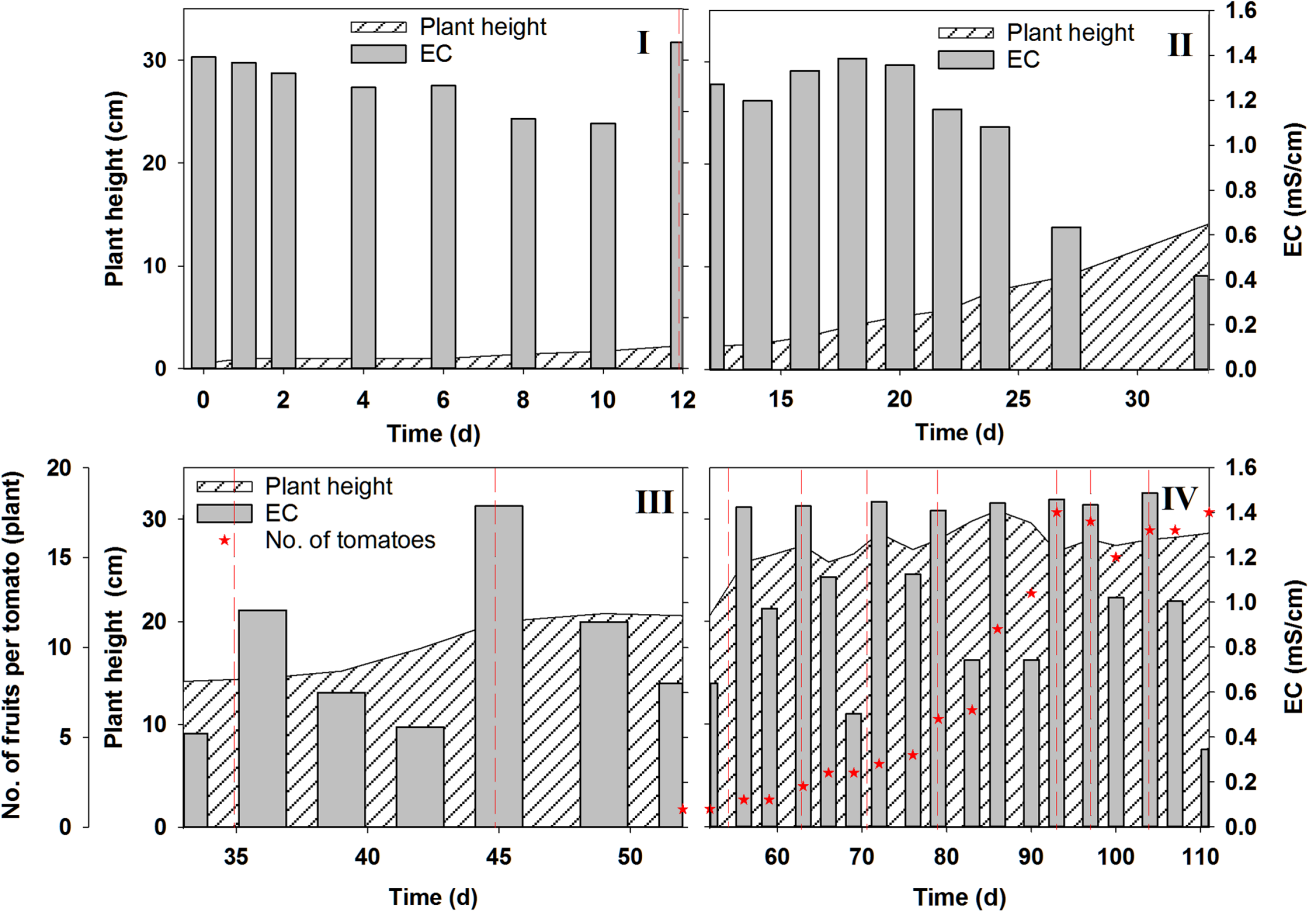
Changes in plant height, electric conductivity (EC), and number of fruits during tomato growth in a closed hydroponic system. Vertical dashed lines indicate when new nutrient solution was added.

Fig 2 (S3 Table) shows the plant height and number of fruits per plant as EC changed during cultivation in four different stages. For the initial 12 d (Stage I: Transplanting), the tomato plants grew little, and the EC was relatively constant before the addition of new nutrient solution at day 12. After day 14 (Stage II: Adaptation), the tomato plants began to grow exponentially and the EC decreased rapidly to below 0.5 mS cm^–1^ at day 33. However, the length of the tomato stems increased little when the EC was low. In Stage III (Flowering), new nutrient solution was added on days 34 and 45, to increase the EC to > 1.2 mS cm^–1^. At this stage, the decrease in EC corresponded to an increase in stem length. The first tomato fruits appeared on day 52 (Stage IV: Fruit set). In Stage IV, new nutrient solution was added several times, indicating increased demand for ions by the plants for fruit set. The length of the tomato stems did not increase greatly; however, the number of fruits increased rapidly, and reached the maximum number at day 93.

### Nutrient uptake rates during tomato growth

**Fig 3:**
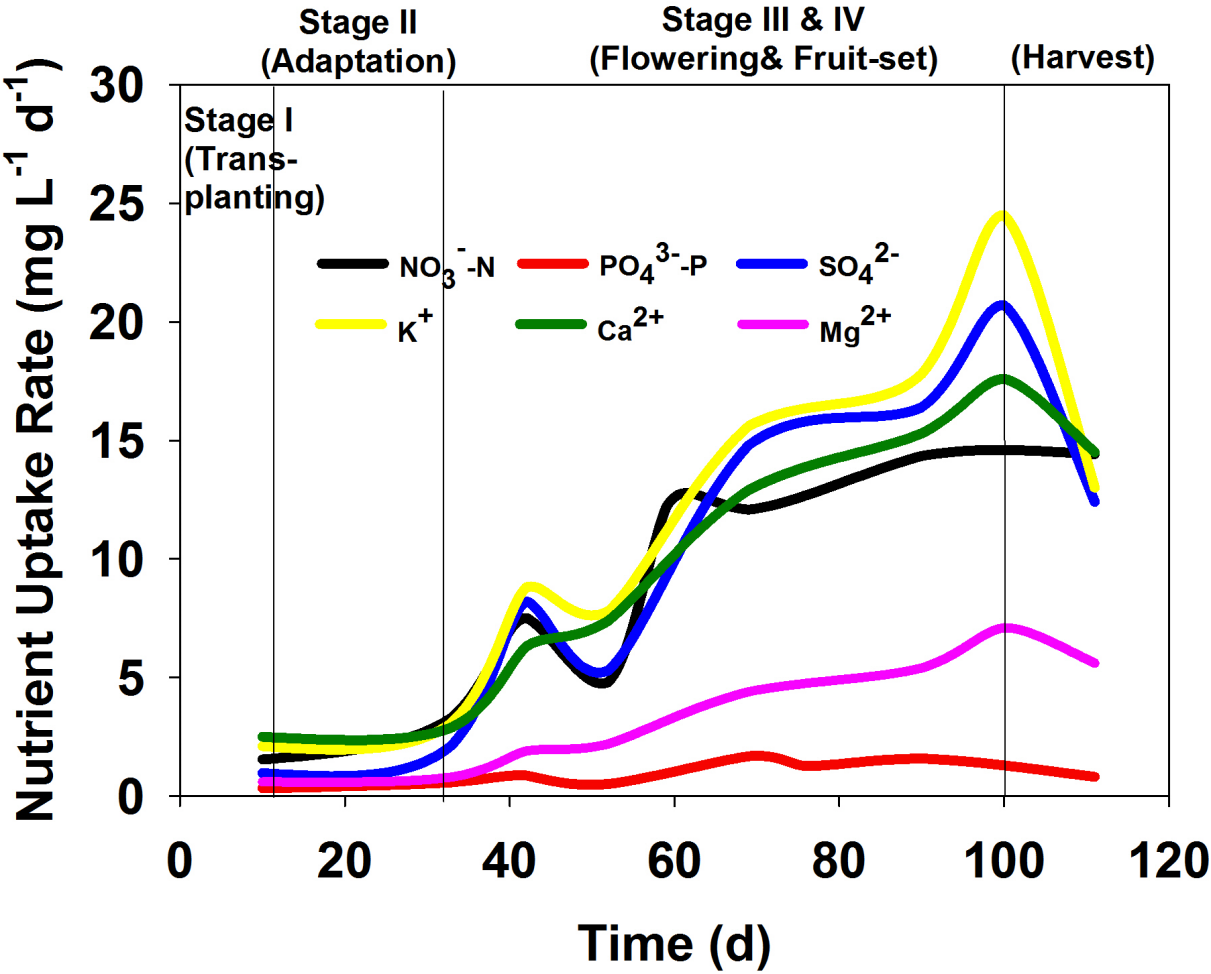
Dynamics of nutrition uptake rates of macro nutrients and secondary nutrients during tomato growth in the closed hydroponic system.

The nutrient uptake rates of major ions during tomato growth were calculated and are shown in Fig 3 (S4 Table). In general, ion uptake rates were in the rank order K^+^ > SO_4_^2–^ > Ca^2+^ > NO_3_^–^-N > Mg^2+^ > PO_4_^3–^-P. In the initial periods (Stage I: Transplanting and Stage II: Adaptation), the uptake rates of all major ions did not increase (< 3 mg L^–1^ d^–1^). When flowering began at day 40 (Stage III: Flowering) and the number of fruits increased (Stage IV: Fruit set), nutrient uptake rates increased significantly until day 100. The maximum uptake rates of K^+^, SO_4_^2–^, Ca^2+^, NO_3_^–^-N, Mg^2+^, and PO_4_^3–^-P were observed to be approximately 25, 20, 17, 13.5, 7, and 1 mg L^–1^ d^–1^, respectively. After fruit harvest, nutrient uptake rates decreased dramatically. The uptake rate of PO_4_^3–^-P was the lowest and varied little compared to that of the other ions.

### Variation in pH, EC, and specific ions during tomato growth

**Fig 4:**
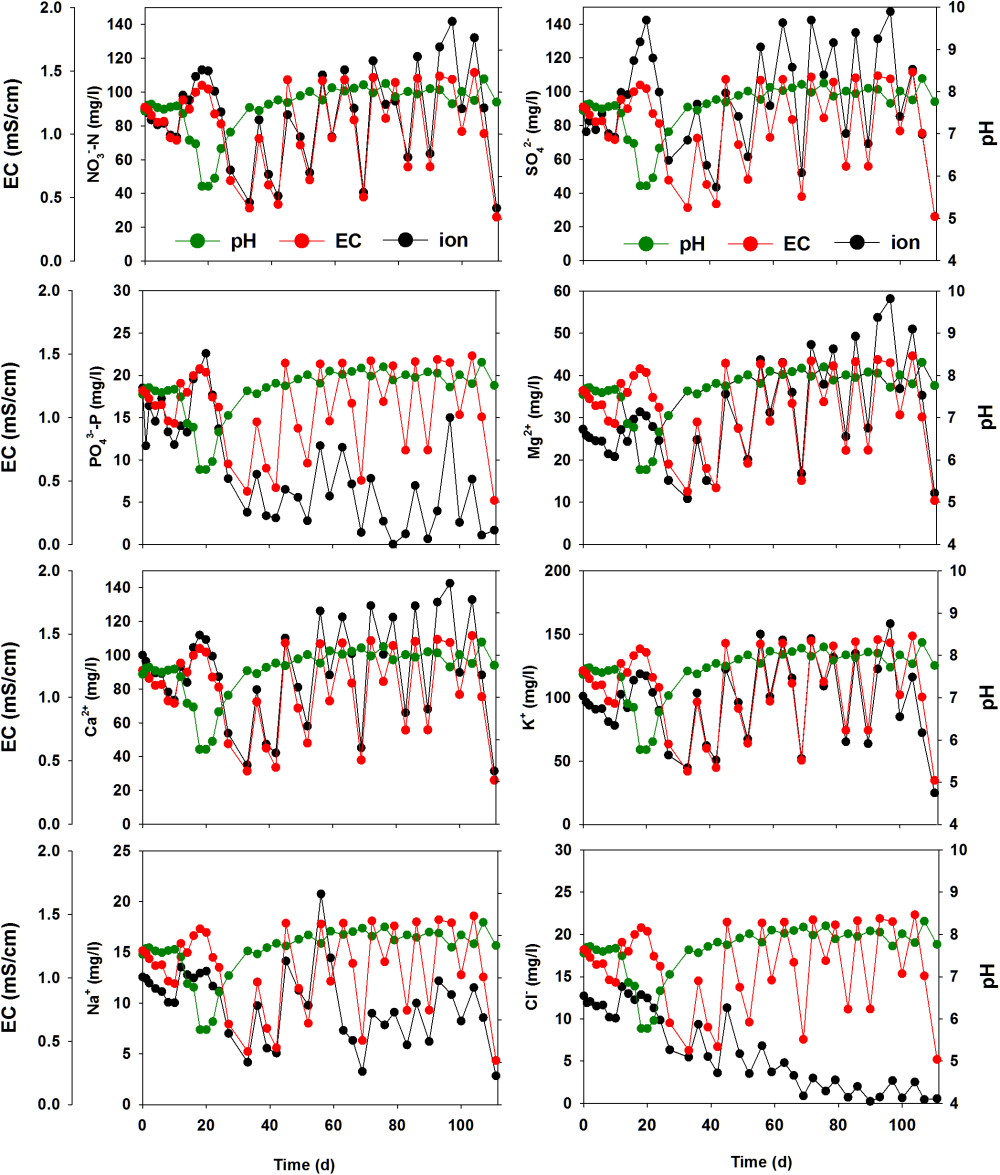
Variation in pH, EC, and major ions (NO_3_^–^-N, PO_4_^3–^-P, Ca^2+^, SO_4_^2–^, K^+^, Mg^2+^, Na^+^, and Cl^–^) during tomato growth in the closed hydroponic system.

**Fig 5:**
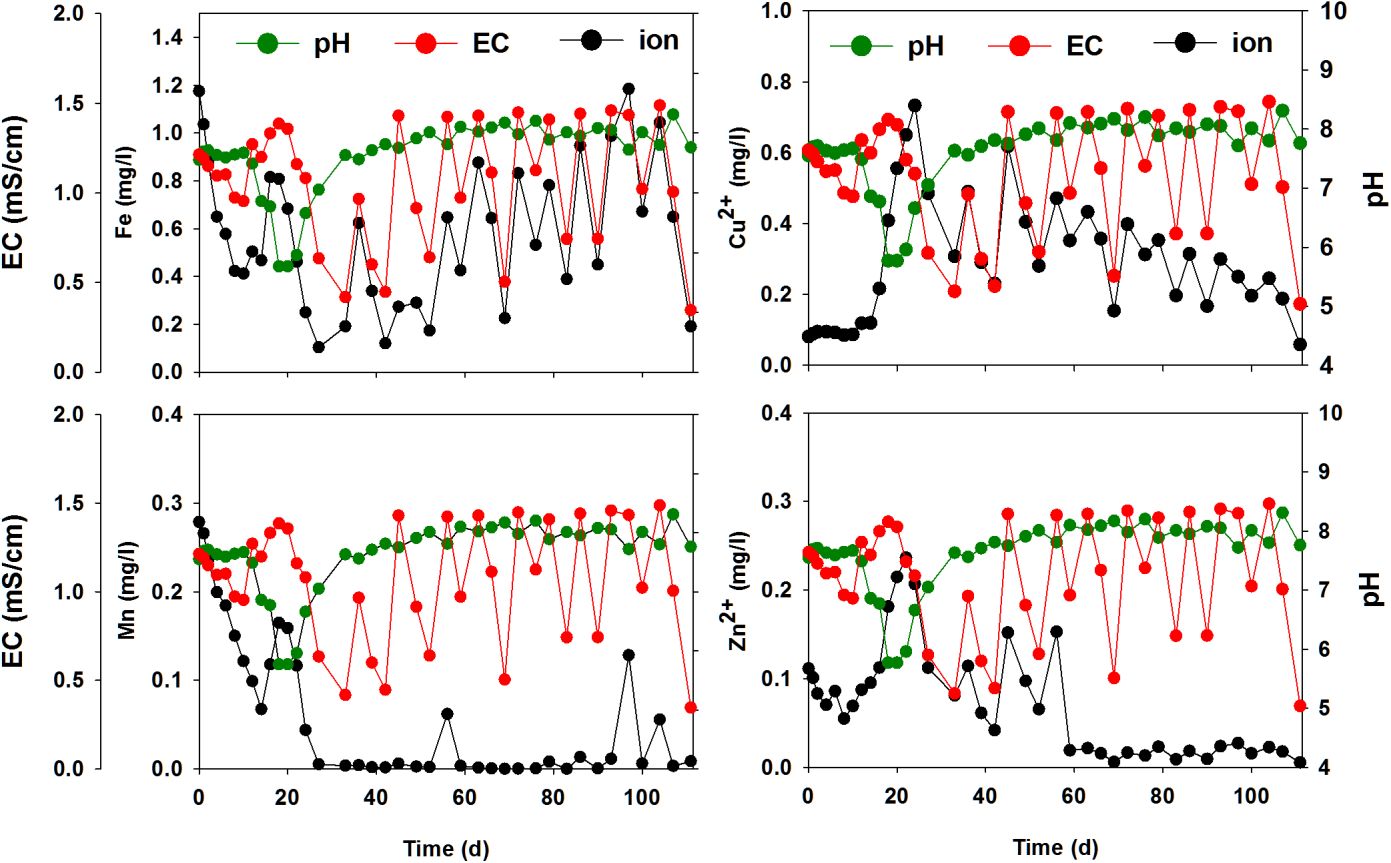
Variation in pH, EC, and minor ions (dissolved Fe, dissolved Mn, Cu^2+^, and Zn^2+^) during tomato growth in the closed hydroponic system.

Variations in pH, EC, and specific major and minor ions during tomato growth were monitored over 120 d and are represented in Figs 4 and 5 (S5 Table). The pH of the nutrient solution ranged between 5.8 and 8.4. The pH decreased during rapid tomato growth. After 12 d and the addition of new nutrient solution, the solution pH decreased rapidly from 7.5 to 5.8, and then increased to 7.5. The pH was then relatively stable until the end of the cultivation period, when it increased slightly to 8.4.

The trends in the variation of NO_3_^–^, SO_4_^2–^, Mg^2+^, and Ca^2+^ concentrations were very similar to that of EC (Fig 4). The concentrations of these specific ions increased or decreased in ways similar to EC. The concentrations of PO_4_^3–^-P, Na^+^, and Cl^−^did not follow the trends in variation of EC (Fig 4). PO_4_^3–^-P concentration initially followed the EC trend, but did not approach its initial concentration (22.6 mg L^–1^) after day 42. In the case of the Na^+^ ion, the trend was similar to the EC trend until day 63; however, the concentration of Na^+^ during the later cultivation period was lower than expected (13.1 mg L^–1^). The trend of Cl^−^variation was clearly different from that of EC. After day 12, the concentration of Cl^−^decreased continuously and approached 0 mg L^–1^.

The variation in concentrations of minor nutrient ions (Fe_diss_, Mn_diss_, Cu^2+^, and Zn^2+^) also differed from that of EC (Fig 5). Fe_diss_ concentrations decreased rapidly from 1.2 mg L^–1^ during the early stages of plant growth, and then followed a trend similar to that of EC in the later stages of cultivation (after day 39). The concentration of Mn_diss_ also declined rapidly and reached almost 0 mg L^–1^ after 25 d. The concentration of Cu^2+^ was low (< 0.1 mg L^–1^) at the beginning of the cultivation and increased with a decrease in pH. Cu^2+^ was lower than expected during later cultivation. The trend of Zn^2+^ concentration was similar to that of Cu^2+^; however, Zn^2+^ concentration rapidly decreased to < 0.02 mg L^–1^ after Day 60.

### Comparison of specific ion concentrations determined by different analytical methods

**Fig 6:**
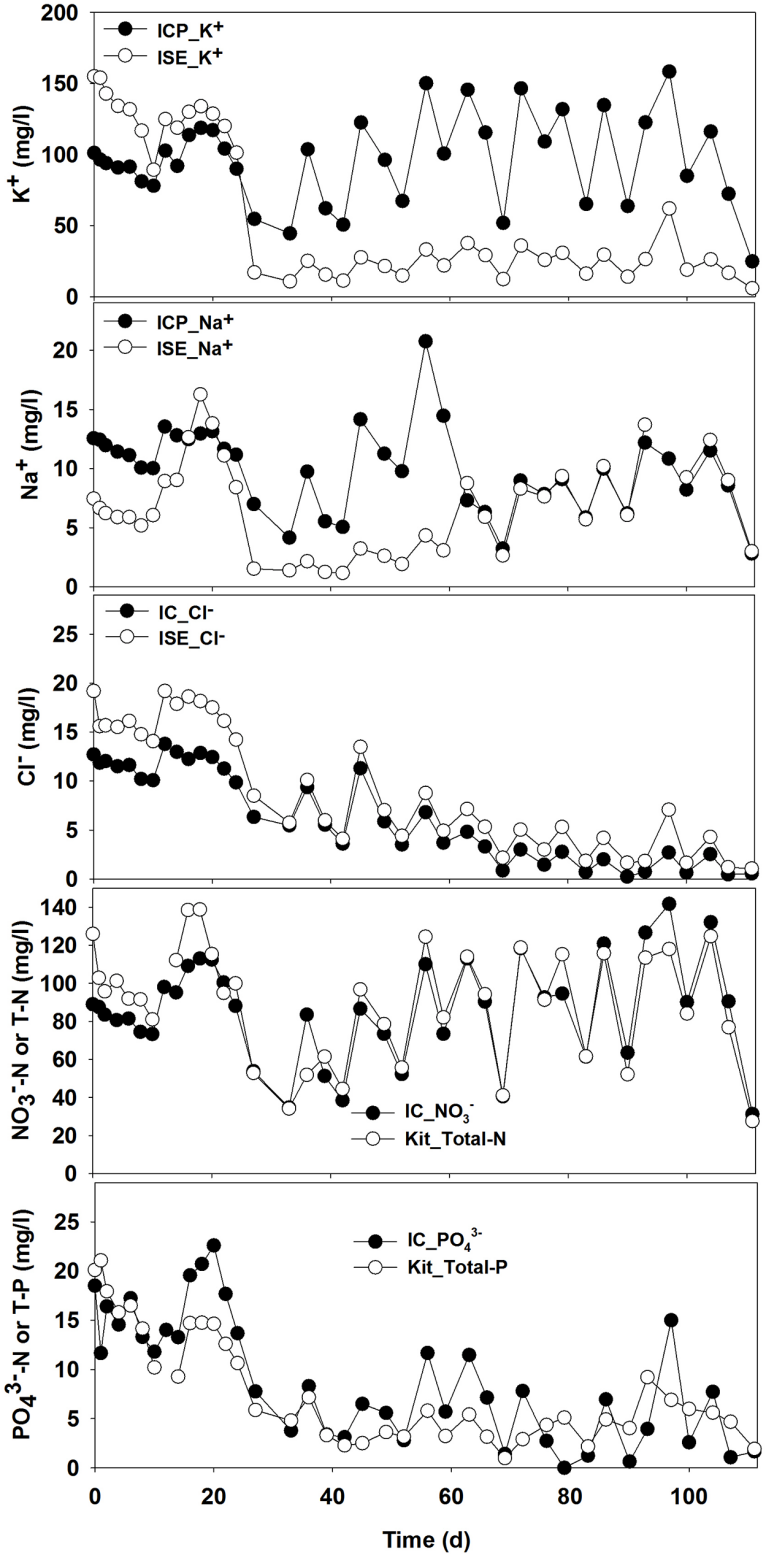
Comparison of ion concentrations determined by analytical instruments [i.e., cations by inductively coupled plasma-optical emission spectroscopy (ICP-OES), anions by ion chromatography (IC)] and on-site measurements [i.e., K^+^, Na^+^, Cl^−^by ion-specific electrodes (ISE) and NO_3_^–^ and PO_4_^3–^ by commercial kit].

Variation in K^+^, Na^+^, Cl^–^, NO_3_^–^-N, and PO_4_^3–^-P concentrations, as determined by established analytical tools such as ICP-OES or IC, were compared with the results from quick on-site assays (ISE/Kit) and are shown in Fig 6 (S6 Table). Variation in the concentrations of NO_3_^–^ over time was similar when measured using two different methods (IC vs. Kit). However, the concentrations of K^+^, Na^+^, Cl^−^and PO_4_^3–^-P measured by two analytical methods (ICP-OES/IC vs. ISE/Kit) were not closely matched for specific time periods.

### Variation of saturation index during tomato growth

**Fig 7:**
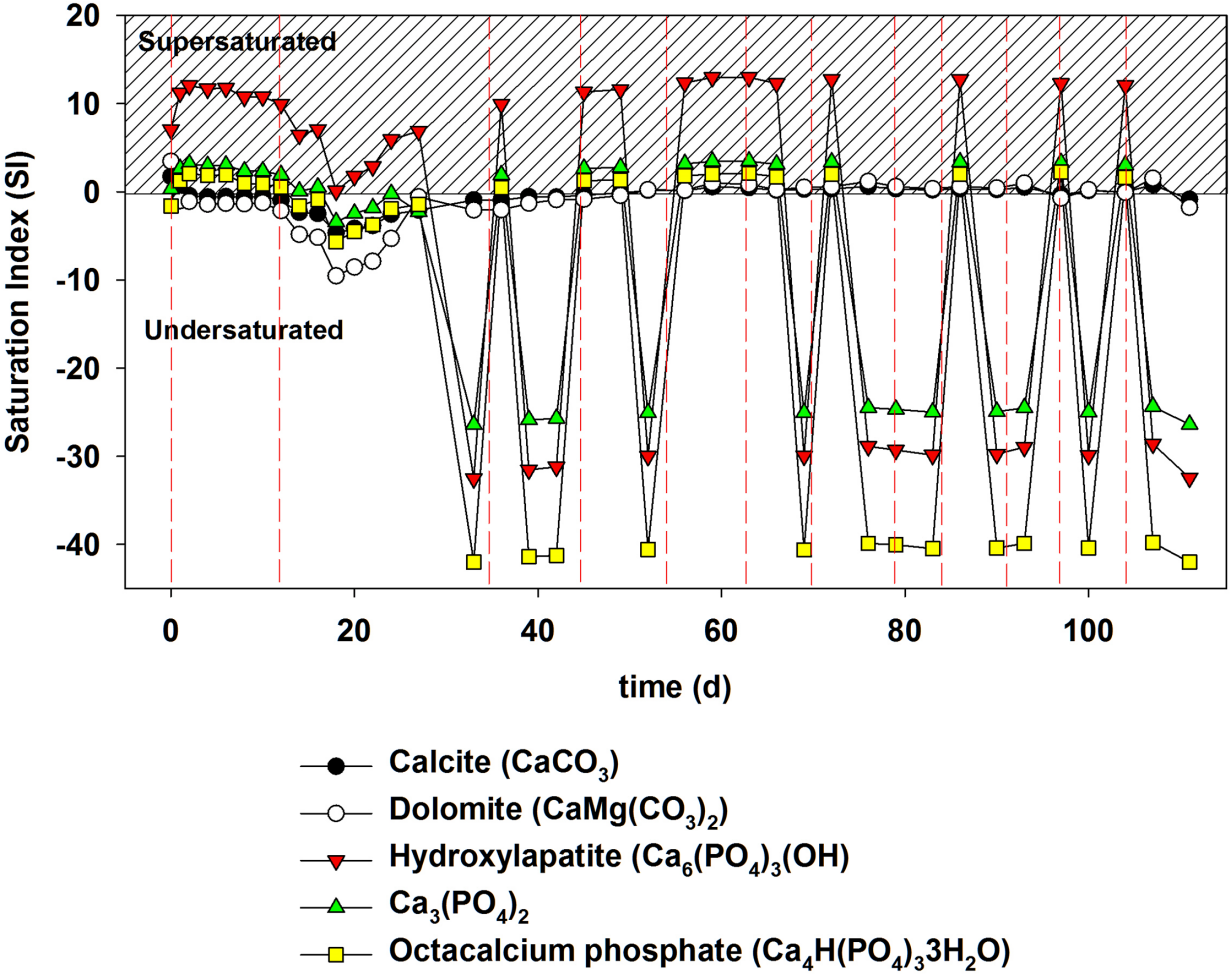
Variation in the saturation index (SI) of several carbonate and phosphate minerals during tomato growth in the closed hydroponic system. Vertical dashed lines indicate when new nutrient solution was added.

To identify the possible minerals precipitated in a closed hydroponic system, the SI was calculated based on the chemical compositions at each time point using MINEQL+. Among many carbonate or phosphate minerals assessed by this modeling technique, mineral phases with SI > 0 at any time points are shown in Fig 7 (S7 Table). The SI of phosphate minerals was higher than that of carbonate minerals.

### Composition and morphology of mineral precipitates

**Fig 8:**
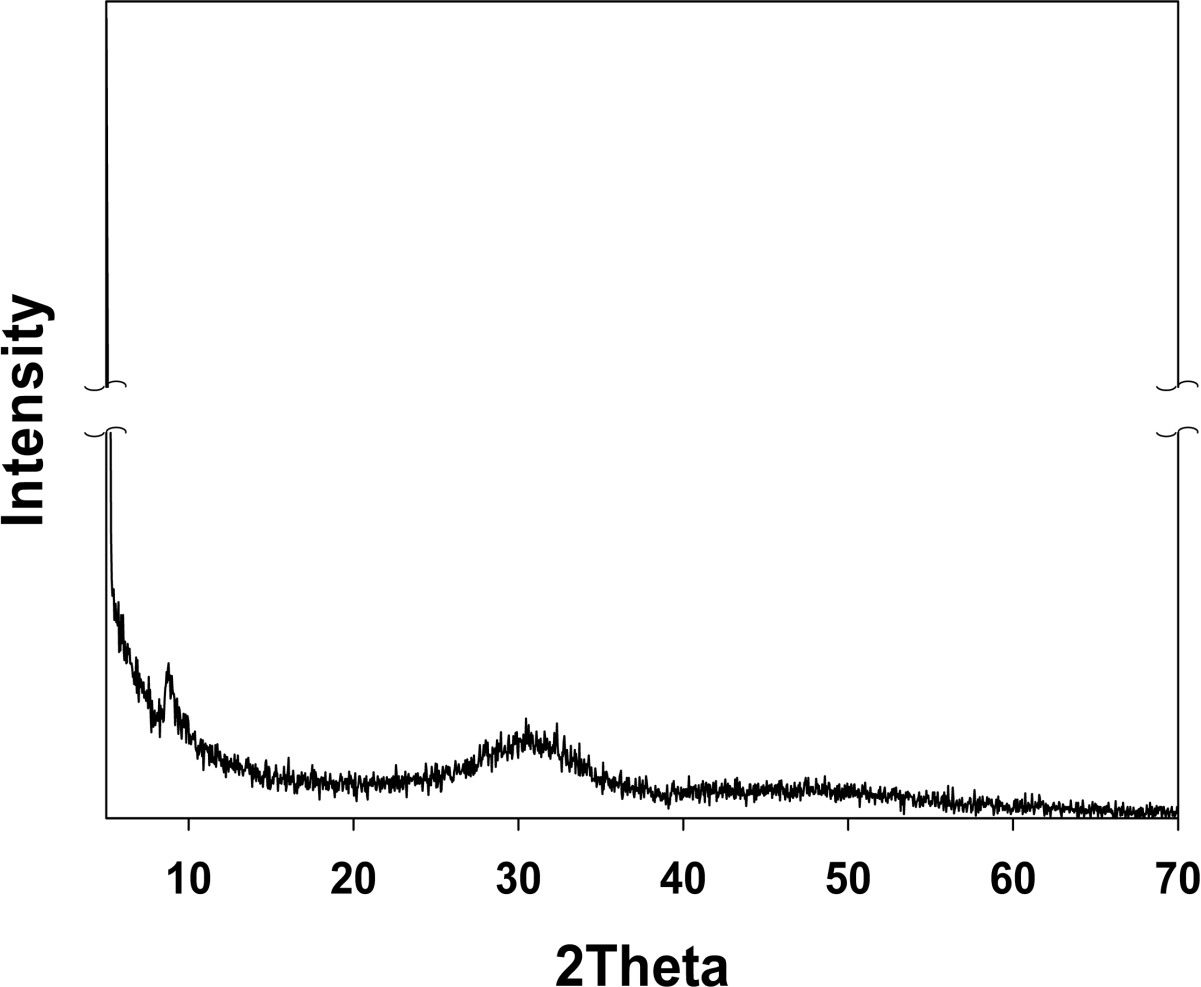
X-ray diffractograms of the precipitates from nutrient solution.

**Fig 9:**
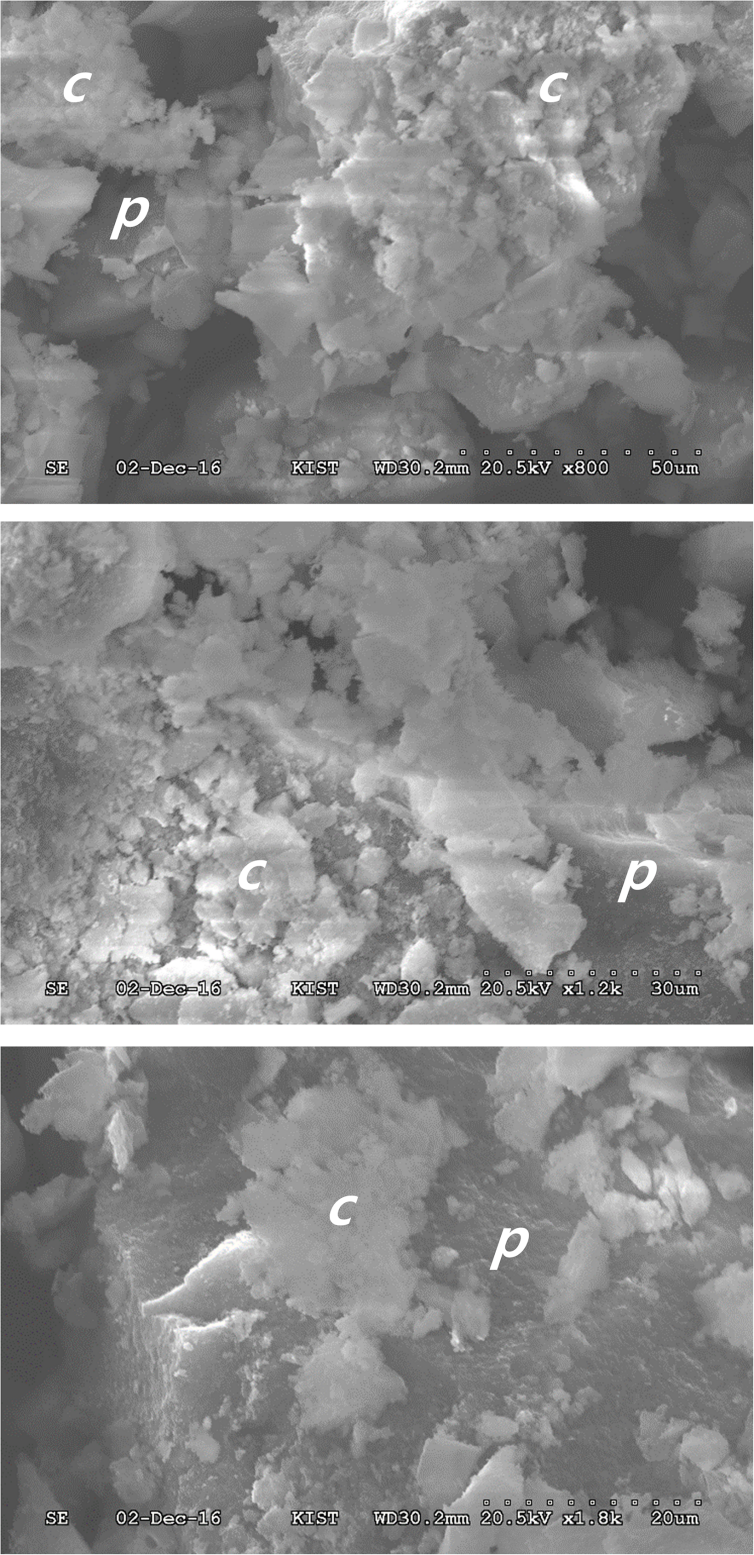
Scanning electron microscope (SEM) photos of the precipitates from nutrient solution. ‘c’ indicates amorphous clusters and ‘p’ indicates plates.

To identify the mineral phases of the precipitates in a closed hydroponic system, we performed additional batch experiments. The result of XRD analysis indicated that the precipitate was amorphous or non-crystalline (Fig 8) and matched with other known phases of calcium phosphate minerals. SEM images showed two different and unique types of morphologies: large plates (*p*) and clusters (*c*) at pH 8.0–8.5 (Fig 9). The Ca/P ratios of the solution and the powder were 1.21 and 1.24, respectively (S8 Table). The analyzed Ca/P ratio (approximately 1.2) for the precipitates formed from the nutrient solution was lower than the standard stoichiometric ratio (1.667) for a pure hydroxyapatite (HA) phase.

## Discussion

### General environmental conditions for tomato growth

The environmental conditions of the indoor closed hydroponic system used in this study were sufficient to support proper tomato cultivation. Tomatoes are known to grow under a wide range of environmental conditions. It has been reported that tomato plants grow in a temperature range of 10–35°C. RH between 30 and 90% is optimal for growth, flowering, fruit set, and fruit growth of tomato plants [16]. In general, the ranges of EC and pH of the nutrient solutions used to grow tomatoes hydroponically are 0.8–2.5 mS cm^–1^ and 5–7, respectively [3]. The environmental conditions of our system were clearly within the optimal ranges; however, nutrient conditions during tomato growth appear not to have been optimal, as we will discuss in the following sections.

### Relationship between EC variation and tomato growth

EC-based nutrient supplementation for plant growth has been widely applied to the closed hydroponic system. The EC decreased with tomato growth over time, but increased again following the addition of new nutrient solutions. Fig 2 shows that when tomato plants absorbed large amounts of dissolved ions (indicated by EC reduction), the plant growth increased. Additionally, during exponential growth, the plants may have used more water than dissolved ions, resulting in an increase in EC (due to concentration). Although EC generally indicates the amount of nutrient ions in the nutrient solution supplied, the uptake rates and extent that specific ions were used by plants is known to vary according to the growth stage [3]. Similar uptake characteristics of EC versus specific ion concentrations were also observed in this study. In this way, physiological disorders in tomato plants can occur due to nutrient deficiency or excess, even under EC-based nutrient control. A variety of nutrients play important roles in determining both fruit yield and fruit quality; thus, a well monitored and regularly adjusted nutrient prescription is essential for cost reduction and the prevention of fertilizer waste.

### Influence of major cations and anions on tomato growth

The amount of the major ions (NO_3_^–^-N, PO_4_^3–^-P, Ca^2+^, SO_4_^2–^, K^+^, Mg^2+^, Na^+^, and Cl^–^) supplied for tomato cultivation was considered to be sufficient based solely on the EC level (1.2–1.5 mS cm^–1^). However, as tomatoes grew, the concentration of each cation and anion in the nutrient solution varied greatly over time (Fig 4). S9 Table represents the Pearson correlation coefficient between EC, pH and major and minor ion concentrations during the tomato plant growth. A strong positive correlation was found between EC and NO_3_^–^, SO_4_^2–^, Ca^2+^, Fe, K^+^, Mg^2+^, Na^+^ concentrations during the plant growth stages. Furthermore, the rates and extent of nutrient uptake by the tomatoes differed for each ion. These results suggest that a specific ion-based control (rather than EC-based control) is essential to improve tomato plant cultivation.

The decrease of pH with rapid tomato growth (Fig 4) may have been related to the decrease in NH_4_^+^ concentration (S2 Fig). The preferential uptake of NH_4_^+^ over NO_3_^–^ by plants at the initial stage of cultivation may have released hydrogen ions (H^+^) from the roots [10], and consequently lowered the pH in the root environment.

The uptake rates of NO_3_^–^-N, K^+^, SO_4_^2–^, and Ca^2+^ were initially slow, but increased rapidly during the flowering stage (Fig 3) indicating that tomato fruits require high amounts of N, K, Ca, and S. The p-value of NO_3_^-^, PO_4_^3-^, SO_4_^2-^, K^+^, Ca^2+^, Mg^2+^ between transplanting and flowering and fruit-set were 0.025, 0.011, 0.010, 0.014, 0.003 and 0.015, respectively which explained the that uptake rate was significant at flowering stages. In fact, Ca^2+^ is an essential nutrient for fruit growth and development. The supply of Ca^2+^ is more critical during the phase of rapid increase in fruit size [5]. Ca^2+^ can also suppress the uptake of Na^+^ as roots preferentially uptake K^+^ over Na^+^ in the presence of sufficient Ca^2+^ [5].

Na^+^ can promote growth and development of plants and improve the flavor of the edible portion [17]. However, the level of Na^+^ was lower than that of EC in this study (Fig 4) indicating that the amount of Na^+^ in the nutrient solution of the experimental hydroponic system was not optimal for tomato growth. Cl^−^is also essential for growth and development of tomatoes and promotes the total and marketable fruit yield [18]. A slightly increased level of Cl^−^can also promote the uptake of cations such as Ca^2+^ by the plant [2]. In this study, the depletion of Cl^−^in the later growth stages (after day 56; Fig 4) suggests that the composition of the experimental nutrient solution did not supply enough Cl^−^for optimal growth of tomatoes, and thus more Cl^−^should be added.

High amounts of P are required for seed formation, growth, fruiting, and fruit quality [19]. Interestingly, the decrease in the amount of PO_4_^3–^-P observed over time in this study was likely due to uptake by the plants and the subsequent precipitation of phosphate mineral caused by changing water quality during tomato growth. In fact, the precipitation of PO_4_^3–^-P, Ca^2+^, Mg^2+^, Fe_diss_, and Mn_diss_ as insoluble minerals at pH > 7, is known to limit nutrient availability for plant uptake [1]. SI values calculated by MINEQL+ modeling also indicated the possible precipitation of hydroxylapatite [Ca_5_(PO_4_)_3_OH], amorphous tricalcium phosphate [ATCP, Ca_3_(PO_4_)_2_], and Ca_4_H(PO_4_)_3_·3H_2_O (Fig 7). Given that the precipitate was amorphous or non-crystalline (Figs 8 and 9) and had a Ca/P ratio of 1.2 (S9 Table), the mineral phase precipitated in this study was most likely Ca_3_(PO_4_)_2_. The composition of amorphous calcium phosphate [ACP, Ca_x_(PO_4_)_y_-nH_2_O] varied, and the atomic ratios of many different ACPs ranged from 1.15 to 1.67 [20]. The formation of octacalcium phosphate [OCP, Ca_8_(HPO_4_)_2_(PO_4_)_4_·5H_2_O] (Ca/P ratio = 1.33) may also have been possible; however, OCP is known to be mostly crystalline [21].

The p-values between each major ion (NO_3_^-^, PO_4_^3-^, SO_4_^2-^, K^+^, Ca^2+^, Mg^2+^) uptake rate during transplanting, flowering and fruit-set stages are reported in S10 and S11 Tables. Results showed that there was no statistical difference between major ion uptake rates during transplanting stages. This clearly indicated that each of major ion consumption during this period was essential for plant growth. During the flowering and fruit-set stages, the PO_4_^3-^ uptake rate has significant difference (p-value <0.05) between other major ions uptake rate (NO_3_^-^, SO_4_^2-^, K^+^, Ca^2+^, Mg^2+^) (S11 Table). This supported the possible phosphorous precipitation observed in batch experiments. In addition, the Ca^2+^ and Mg^2+^ ion uptake rate had also p-value of 0.022 explaining the possible precipitation of either Ca^2+^ or Mg^2+.^

### Impact of minor nutrients on tomato growth

Supplying minor nutrients (i.e., Fe_diss_, Mn_diss_, Cu^2+^, and Zn^2+^) is essential for high yield potential in tomato. Minor nutrients are required for better fruit chemical composition and general plant condition [1, 5, 22]. As discussed above, the decrease in Fe_diss_ may have been due to the increase of the pH of the nutrient solution. The optimal pH for Fe-EDTA (ethylenediaminetetraacetate) is within the range of 4.0–6.5; however, Fe-EDTA in a nutrient solution at pH 7.0 could become 25% insoluble [23]. A lack of Fe_diss_ in the nutrient solution was also due to the breakdown of chelated Fe by UV light. The chelated iron, a soluble complex of iron and chelating agents such as EDTA, can be decomposed quickly by various wavelengths of light including visible and UV [24, 25]. The rapid decrease in Fe_diss_ and Mn_diss_ suggests that LED lighting may have degraded Fe/Mn-chelates (Fig 5), subsequently resulting in the precipitation of Fe and Mn. At the early stage, the nutrient solution could absorb LED radiation over a wide surface area because the tomato plants initially grew very little. However, when the plants later grew exponentially, self-shading within the canopy and mutual shading between plants may have blocked LED light to the nutrient solution surface. Therefore, the Fe-chelates may not have been decomposed by light.

Unlike those of the other nutrient ions, the initial concentrations of Cu^2+^ and Zn^2+^ were low and relatively constant before day 18 (Fig 5), which is likely not due to plant uptake. Rather, the results suggest that Cu^2+^ and Zn^2+^ might be sorbed onto and initially co-precipitated with phosphate mineral precipitates. The immobilization and removal of Zn and Cu by phosphate minerals has been reported in previous studies [26–28]. Cu^2+^ and Zn^2+^ increased to maximum levels with a decrease in pH due to acidic dissolution of these ions from the mineral precipitates. Compared with EC levels, the Mn_diss_, Cu^2+^, and Zn^2+^ appear to have been depleted in the later periods of tomato cultivation, suggesting that more of these ions should be supplied during these periods.

### Limitation of determining concentrations of major ions using quick on-site assay

To confirm whether currently available rapid assays or measurement techniques for several ions are useful for specific ion monitoring, we extensively monitored and compared several ions (Fig 6). The concentrations of K^+^ and Na^+^ determined by ion-specific electrodes were not comparable with those determined by ICP-OES. S12 Table illustrates the p-value determination by performing two-sample t-test to evaluate the statistical difference between various analytical measurements of K^+^, Na^+^, Cl^–^, NO_3_^–^ and PO_4_^3–^ concentrations. Results indicated that K^+^, Na^+^ and Cl^−^concentrations measurement were statistically different using ISE and ICP techniques. In addition, there was no positive correlation between those two techniques for K^+^ and Na^+^ except Cl^−^(S12 Table). The measurement of NO_3_^–^ and PO_4_^3–^ concentrations using Kit and ICP exhibited no statistical difference (p-value > 0.05) and had positive correlation (R^2^ ≥ 0.65). Inconsistent results for several ion concentrations determined by different analytical methods suggest interference with other ions and experimental conditions when using the quick on-site assay (ISE/Kit) during measurement of any specific compound. Therefore, careful interpretation of the results from the quick on-site assay and the further development of a new ISE and colorimetric assay are essential for more accurate, rapid on-site assays of specific ions. Rapid analytical methods are particularly needed for Na^+^, Cl^–^, PO_4_^3–^, and SO_4_^2–^, among the major ions, because their concentrations are not likely to match EC trends (Fig 4). Additionally, better and reliable detection methods for K^+^, Na^+^, and Cl^−^should be developed because the current rapid assays for these ions are not reliable compared to conventional instrumental analysis (Fig 6).

### Implications of cation and anion measurement for efficient crop production

Extensive monitoring of each ion in nutrient solution during tomato plant growth showed that some ions were unexpectedly depleted over time. Such depletion may be due to preferential uptake during specific growth stages; however, equilibrium modeling and the mineral precipitation experiment also showed the potential for precipitation of phosphate minerals and subsequent sorptive removal of trace-metals from solution. The results suggest that an EC-based monitoring strategy might provide insufficient and incorrect information on nutrient supply to the target plant. Therefore, the concentration of each ion (at least the major ions) should be monitored to achieve successful outcomes for plant and crop cultivation in hydroponic systems.

## Conclusions

The results of this study suggest that to improve crop cultivation it is necessary to monitor ion deficiency regularly during crop growth in a ‘closed’ hydroponic system. The key findings of this study are listed below:

1. Major cations and anions measurement in nutrient solution during tomato growth suggested that EC-based farming systems are not efficient for crop production due to imbalance and deficiency of specific ions.
2. To achieve the maximum fruit yield, it is necessary to supply major ions (i.e., PO_4_^3–^, and Cl^–^) for plant growth.
3. In addition to major ions, some minor nutrients (i.e., dissolved Mn, Cu^2+^, and Zn^2+^) are also required as catalysts for plant growth.
4. Phosphate mineral precipitation facilitated abiotic removal of dissolved Fe, dissolved Mn, Cu^2+^, and Zn^2+^.
5. The concentrations of T-N and T-P determined by the commercial kits were relatively reliable, but K^+^, Na^+^, and Cl^−^determined by the ion specific electrodes had variable results with ICP-OES. This observation demonstrates that there is a need to further develop a specific electrode for the immediate assessment of the specific nutrients in actual farms.

This extensive study of nutrient uptake characterization during tomato growth may provide information and insight for other types of vegetable or fruit farming to improve our understanding of nutrient dynamics, imbalance, and deficiency.

## Supporting information

S1 Fig Temporal variation in atmospheric and nutrient solution conditions in the indoor farming system during tomato growth. (TIF)

S2 Fig Variation in pH, electric conductivity (EC), and ammonium (NH4+) during tomato growth in the closed hydroponic system. Vertical dashed lines indicate when new nutrient solution was added.
(TIF)

S1 Table The chemical composition of nutrient solution. (DOCX)

S2 Table Detection limits and instrument calibration ranges. (DOCX)

S3 Table Changes in plant height, electric conductivity (EC), and number of fruits during tomato growth in a closed hydroponic system. (DOCX)

S4 Table Dynamics of nutrition uptake rates of macro nutrients and secondary nutrients during tomato growth in the closed hydroponic system (DOCX)

S5 Table Variation in pH, EC, and major/minor ions during tomato growth in the closed hydroponic system. (DOCX)

S6 Table Comparison of ion concentrations determined by analytical instruments [i.e., cations by inductively coupled plasma-optical emission spectroscopy (ICP-OES), anions by ion chromatography (IC)] and on-site measurements [i.e., K^+^, Na^+^, Cl^−^by ion-specific electrodes (ISE) and NO_3_^–^ and PO_4_^3–^ by commercial kit]. (DOCX)

S7 Table Variation in the saturation index (SI) of several carbonate and phosphate minerals during tomato growth in the closed hydroponic system. (DOCX)

S8 Table Ca/P ratio, morphology, and mineralogy of solid particles precipitated from nutrient solution. (DOCX)

S9 Table Pearson correlation coefficient determination between electrical conductivity, pH and major and minor cations and anions concentration during the tomato plant growth. (DOCX)

S10 Table Two-sample t-test (unequal variances) for p-value determination (significance level of 5% or α of 0.05) between major nutrients uptake rate (mg L^–1^ d^–1^) at transplanting phase of plants growth. (DOCX)

S11 Table Two-sample t-test (unequal variances) for p-value determination (significance level of 5% or α of 0.05) between major nutrients uptake rate (mg L^–1^ d^–1^) at flowering and fruit-set phases of tomato growth. (DOCX)

S12 Table Two-sample t-test (unequal variances) for p-value (significance level of 5% or α of 0.05) and linear regression coefficient determination between ion concentrations determined by various analytical instruments [i.e., cations by inductively coupled plasma-optical emission spectroscopy (ICP-OES), anions by ion chromatography (IC)] and on-site measurements [i.e., K^+^, Na^+^, Cl^−^by ion-specific electrodes (ISE) and NO_3_^–^ and PO_4_^3–^ by commercial kit]. (DOCX)
