# Supplementary material for: Characterizing nutrient uptake kinetics for efficient crop production during Solanum lycopersicum var. cerasiforme Alef. growth in a closed indoor hydroponic system
Source: PLoS One. 2017 May 9;12(5):e0177041. doi: 10.1371/journal.pone.0177041 (PMC5423622; doi:10.1371/journal.pone.0177041)
Supplement: S1 Table — (DOCX) [file pone.0177041.s003.docx]

S1 Table. The chemical composition of nutrient solution.

|  | **MW** | **Concentrations** | |
| --- | --- | --- | --- |
|  | g mol^-1^ | mg L^-1^ | mmol L^-1^ |
| **Major Elements** | | | |
| KNO_3_ | 101.10 | 83 | 0.82 |
| Ca(NO_3_)_2_ - 4H_2_O | 236.15 | 473 | 2.00 |
| MgSO_4_ - 7H_2_O | 246.47 | 248 | 1.01 |
| NH_4_H_2_PO_4_ | 115.03 | 40 | 0.35 |
| KH_2_PO_4_ | 136.09 | 68 | 0.50 |
| **Trace Elements** | | | |
| Fe-EDTA | 348.09 | 10 | 0.03 |
| H_3_BO_3_ | 61.83 | 1.25 | 0.020 |
| MnSO_4_ - 4H_3_O | 227.09 | 1.0 | 0.0044 |
| ZnSO_4_ - 7H_2_O | 287.53 | 0.10 | 3.48E-04 |
| CuSO_4_ - 5H_2_O | 249.69 | 0.10 | 4.01E-04 |
| NaMoO_4_ - 2H_2_O | 241.95 | 0.03 | 1.24E-04 |
