# Supplementary material for: Characterizing nutrient uptake kinetics for efficient crop production during Solanum lycopersicum var. cerasiforme Alef. growth in a closed indoor hydroponic system
Source: PLoS One. 2017 May 9;12(5):e0177041. doi: 10.1371/journal.pone.0177041 (PMC5423622; doi:10.1371/journal.pone.0177041)
Supplement: S2 Table — (DOCX) [file pone.0177041.s004.docx]

S2 Table. Detection limits and instrument calibration ranges.

| Analyte | Method | Detection limit  (mg kg^-1^) | Calibration range (mg kg^-1^) |
| --- | --- | --- | --- |
| NO_3_^–^-N | IC | 0.10 | 0.1~20 |
| PO_4_^3–^-P | IC | 0.10 |  |
| SO_4_^2–^ | IC | 0.10 |  |
| Cl^–^ | IC | 0.10 |  |
| Ca^2+^ | ICP-OES | 0.50 | 0.5~20 |
| Na^+^ | ICP-OES | 0.50 |  |
| K^+^ | ICP-OES | 0.50 |  |
| Mg^2+^ | ICP-OES | 0.50 |  |
| Dissolved Fe | ICP-OES | 0.50 |  |
| Dissolved Mn | ICP-OES | 0.50 |  |
| Dissolved Si | ICP-OES | 0.50 |  |
| Zn^2+^ | ICP-OES | 1.00 |  |
| Cu^2+^ | ICP-OES | 1.00 |  |
| Total N | KIT | 0.06 | 0.06~3.5 |
| Total P | KIT | 3 | 3~25 |
| K^+^ | ISE | 39 | 150~2000 |
| Na^+^ | ISE | 23 | 150~2000 |
| Ca^2+^ | ISE | 40 | 150~2000 |
