# Supplementary material for: Characterizing nutrient uptake kinetics for efficient crop production during Solanum lycopersicum var. cerasiforme Alef. growth in a closed indoor hydroponic system
Source: PLoS One. 2017 May 9;12(5):e0177041. doi: 10.1371/journal.pone.0177041 (PMC5423622; doi:10.1371/journal.pone.0177041)
Supplement: S3 Table — (DOCX) [file pone.0177041.s005.docx]

S3 Table. Changes in plant height, electric conductivity (EC), and number of fruits during tomato growth in a closed hydroponic system.

| Time (d) | EC (mS cm^-1^) | Tomato fruit  number | Plant length (cm) |
| --- | --- | --- | --- |
| 0 | 1.21 |  | 0.5 |
| 1 | 1.19 |  | 1.02 |
| 2 | 1.15 |  | 1 |
| 4 | 1.09 |  | 0.96 |
| 6 | 1.10 |  | 1 |
| 8 | 0.97 |  | 1.42 |
| 10 | 0.95 |  | 1.7 |
| 12 | 1.27 |  | 2.26 |
| 14 | 1.20 |  | 2.5 |
| 16 | 1.33 |  | 3.2 |
| 18 | 1.39 |  | 4.3 |
| 20 | 1.36 |  | 5.18 |
| 22 | 1.16 |  | 5.8 |
| 24 | 1.08 |  | 7.7 |
| 27 | 0.63 |  | 9.1 |
| 33 | 0.42 |  | 14.2 |
| 36 | 0.97 |  | 14.5 |
| 39 | 0.60 |  | 15.2 |
| 42 | 0.45 |  | 17.4 |
| 45 | 1.43 |  | 20 |
| 49 | 0.91 |  | 20.8 |
| 52 | 0.64 | 1 | 20.6 |
| 56 | 1.42 | 1.5 | 25.8 |
| 59 | 0.97 | 1.5 | 26.4 |
| 63 | 1.43 | 2.25 | 27.4 |
| 66 | 1.11 | 3 | 25.8 |
| 69 | 0.50 | 3 | 26.6 |
| 72 | 1.45 | 3.5 | 28.6 |
| 76 | 1.12 | 4 | 27 |
| 79 | 1.41 | 6 | 28 |
| 83 | 0.74 | 6.5 | 29.8 |
| 86 | 1.44 | 11 | 30.8 |
| 90 | 0.74 | 13 | 29.6 |
| 93 | 1.46 | 17.5 | 26.8 |
| 97 | 1.43 | 17 | 28 |
| 100 | 1.02 | 15 | 27.4 |
| 104 | 1.49 | 16.5 | 28 |
| 107 | 1.00 | 16.5 | 28.2 |
| 111 | 0.35 | 17.5 | 28.6 |
