# Supplementary material for: Characterizing nutrient uptake kinetics for efficient crop production during Solanum lycopersicum var. cerasiforme Alef. growth in a closed indoor hydroponic system
Source: PLoS One. 2017 May 9;12(5):e0177041. doi: 10.1371/journal.pone.0177041 (PMC5423622; doi:10.1371/journal.pone.0177041)
Supplement: S4 Table — (DOCX) [file pone.0177041.s006.docx]

S4 Table. Dynamics of nutrition uptake rates of macro nutrients and secondary nutrients during tomato growth in the closed hydroponic system

| t (d) | Nutrient uptake rates (mg L^-1^ d^-1^) | | | | | | | | | | | | | | |
| --- | --- | --- | --- | --- | --- | --- | --- | --- | --- | --- | --- | --- | --- | --- | --- |
|  | NO_3_^-^ | PO_4_^3-^ | SO_4_^2-^ | Cl^-^ | NO_2_^-^ | Ca^2+^ | K^+^ | Mg^2+^ | Na^+^ | Dissolved  Fe | Dissolved  Mn | Dissolved  Si | Cu^2+^ | Zn^2+^ | NH_4_^+^ |
| 10 | 1.5 | 0.3 | 1.0 | 0.2 | 0 | 2.5 | 2.1 | 0.6 | 0.3 | 0.1 | 0 | 0.1 | 0 | 0 | 0 |
| 33 | 3.3 | 0.6 | 2.2 | 0.4 | 0.4 | 2.9 | 3.1 | 0.8 | 0.4 | 0 | 0 | 0.1 | 0 | 0 | 0 |
| 42 | 7.5 | 0.9 | 8.2 | 1.0 | 0.2 | 6.3 | 8.8 | 1.9 | 0.8 | 0.1 | 0 | 0 | 0 | 0 | 0 |
| 52 | 4.8 | 0.5 | 5.3 | 1.1 | 0 | 7.4 | 7.8 | 2.2 | 0.6 | 0 | 0 | 0.3 | 0 | 0 | 0 |
| 59 | 12.2 | - | - | - | - | - | - | - | - | - | - | - | - | - | - |
| 69 | 12.1 | 1.7 | 14.8 | 0.7 | 0 | 12.9 | 15.6 | 4.4 | 0.7 | 0.1 | 0 | 0.7 | 0 | 0 | 0 |
| 76 | - | 1.3 | - | 0.4 | - | - | - | - | - | 0.1 | - | - | 0 | - | - |
| 90 | 14.3 | 1.6 | 16.4 | 0.7 | 0 | 15.3 | 17.8 | 5.4 | 0.9 | 0.1 | 0 | 0.9 | 0 | 0 | 0 |
| 100 | - | 4.1 | 20.7 |  | 0 | 17.6 | 24.5 | 7.1 | 0.9 | 0.2 | 0 | 0.9 | 0 | 0 | 0 |
| 111 | 14.4 | 0.8 | 12.4 | 0.3 | 0 | 14.5 | 13 | 5.6 | 1.3 | 0.1 | 0 | 1.2 | 0 | 0 | 0.1 |
