# Supplementary material for: Characterizing nutrient uptake kinetics for efficient crop production during Solanum lycopersicum var. cerasiforme Alef. growth in a closed indoor hydroponic system
Source: PLoS One. 2017 May 9;12(5):e0177041. doi: 10.1371/journal.pone.0177041 (PMC5423622; doi:10.1371/journal.pone.0177041)
Supplement: S5 Table — (DOCX) [file pone.0177041.s007.docx]

S5 Table. Variation in pH, EC, and major/minor ions during tomato growth in the closed hydroponic system.

| Sampling point | Nutrient soln. inj. time | EC | pH | Concentrations (mg L^-1^) | | | | | | | | | | | | | |
| --- | --- | --- | --- | --- | --- | --- | --- | --- | --- | --- | --- | --- | --- | --- | --- | --- | --- |
|  |  |  |  | NO_3_-N | PO_4_-P | SO4 | Cl | Ca | Cu | Fe | K | Mg | Na | Si | Zn | Mn | NH4 |
| 0 | 0 | 1.21 | 7.55 | 89.0 | 18.5 | 89.8 | 12.7 | 100.0 | 0.1 | 1.2 | 101.1 | 27.2 | 12.6 | 4.8 | 0.1 | 0.3 | 2.0 |
| 1 |  | 1.19 | 7.69 | 87.5 | 11.7 | 76.2 | 11.9 | 96.3 | 0.1 | 1.0 | 96.4 | 25.8 | 12.4 | 4.7 | 0.1 | 0.3 | 2.1 |
| 2 |  | 1.15 | 7.71 | 83.5 | 16.4 | 82.5 | 12.0 | 93.0 | 0.1 | 0.9 | 93.9 | 25.3 | 12.0 | 4.5 | 0.1 | 0.2 | 2.0 |
| 4 |  | 1.09 | 7.63 | 80.7 | 14.6 | 77.5 | 11.5 | 89.4 | 0.1 | 0.6 | 90.8 | 24.5 | 11.4 | 4.4 | 0.1 | 0.2 | 1.9 |
| 6 |  | 1.10 | 7.59 | 81.5 | 17.2 | 86.9 | 11.6 | 89.1 | 0.1 | 0.6 | 91.4 | 24.4 | 11.1 | 4.3 | 0.1 | 0.2 | 1.9 |
| 8 |  | 0.97 | 7.64 | 74.5 | 13.3 | 75.0 | 10.2 | 78.2 | 0.1 | 0.4 | 81.1 | 21.4 | 10.1 | 3.8 | 0.1 | 0.2 | 1.7 |
| 10 |  | 0.95 | 7.67 | 73.3 | 11.8 | 73.0 | 10.1 | 73.3 | 0.1 | 0.4 | 78.0 | 20.8 | 10.0 | 3.5 | 0.1 | 0.1 | 1.6 |
| 12 | 12 | 1.27 | 7.49 | 98.1 | 14.0 | 99.5 | 13.8 | 93.3 | 0.1 | 0.5 | 102.7 | 27.2 | 13.5 | 3.8 | 0.1 | 0.1 | 1.9 |
| 14 |  | 1.20 | 6.86 | 95.2 | 13.3 | 98.2 | 13.0 | 83.8 | 0.1 | 0.5 | 92.0 | 24.4 | 12.8 | 2.3 | 0.1 | 0.1 | 1.1 |
| 16 |  | 1.33 | 6.77 | 109.2 | 19.6 | 118.3 | 12.2 | 104.5 | 0.2 | 0.8 | 113.7 | 29.6 | 12.5 | 2.0 | 0.1 | 0.1 | 0.8 |
| 18 |  | 1.39 | 5.77 | 113.0 | 20.7 | 129.4 | 12.9 | 111.9 | 0.4 | 0.8 | 118.8 | 31.4 | 12.9 | 1.4 | 0.2 | 0.2 | 0.8 |
| 20 |  | 1.36 | 5.77 | 112.5 | 22.6 | 142.2 | 12.4 | 109.3 | 0.6 | 0.7 | 117.2 | 30.4 | 13.1 | 1.2 | 0.2 | 0.2 | 0.5 |
| 22 |  | 1.16 | 5.96 | 100.4 | 17.7 | 119.8 | 11.3 | 99.5 | 0.6 | 0.5 | 104.1 | 27.9 | 11.7 | 0.9 | 0.2 | 0.1 | 0.3 |
| 24 |  | 1.08 | 6.66 | 88.1 | 13.7 | 99.7 | 9.9 | 87.2 | 0.7 | 0.2 | 90.0 | 24.6 | 11.2 | 0.9 | 0.2 | 0.0 | 0.1 |
| 27 |  | 0.63 | 7.05 | 53.8 | 7.8 | 59.3 | 6.3 | 53.8 | 0.5 | 0.1 | 54.7 | 15.1 | 7.0 | 0.6 | 0.1 | 0.0 | 0.0 |
| 33 |  | 0.42 | 7.63 | 34.7 | 3.8 | 71.2 | 5.5 | 34.9 | 0.3 | 0.2 | 44.5 | 10.8 | 4.2 | 0.6 | 0.1 | 0.0 | 0.0 |
|  | 34(10/1) | 1.57 |  | 114.7 | 23.9 | 115.8 | 15.4 | 128.9 | 0.7 | 1.5 | 130.3 | 35.1 | 16.2 | 6.2 | 0.2 | 0.4 | 2.57 |
| 36 |  | 0.97 | 7.56 | 83.5 | 8.3 | 92.6 | 9.4 | 79.6 | 0.5 | 0.6 | 103.6 | 24.8 | 9.7 | 1.5 | 0.1 | 0.0 | 0.0 |
| 39 |  | 0.60 | 7.71 | 51.3 | 3.4 | 56.3 | 5.5 | 47.3 | 0.3 | 0.3 | 62.1 | 15.1 | 5.5 | 1.4 | 0.1 | 0.0 | 0.0 |
| 42 |  | 0.45 | 7.81 | 38.5 | 3.1 | 43.4 | 3.6 | 42.1 | 0.2 | 0.1 | 50.6 | 13.4 | 5.1 | 1.3 | 0.0 | 0.0 | 0.0 |
| 45 | 45 | 1.43 | 7.75 | 86.6 | 6.5 | 99.2 | 11.3 | 110.1 | 0.6 | 0.3 | 122.6 | 35.6 | 14.1 | 4.8 | 0.2 | 0.0 | 0.1 |
| 49 |  | 0.91 | 7.91 | 73.4 | 5.6 | 85.2 | 5.9 | 80.9 | 0.4 | 0.3 | 96.2 | 27.5 | 11.3 | 3.6 | 0.1 | 0.0 | 0.1 |
| 52 |  | 0.64 | 8.01 | 52.3 | 2.8 | 61.4 | 3.5 | 57.9 | 0.3 | 0.2 | 67.3 | 20.1 | 9.8 | 2.6 | 0.1 | 0.0 | 0.1 |
|  | 54(10/24) | 1.43 |  | 105.2 | 21.9 | 106.1 | 15.0 | 118.2 | 0.6 | 1.4 | 119.5 | 32.1 | 14.6 | 5.7 | 0.2 | 0.4 | 2.36 |
| 56 |  | 1.42 | 7.81 | 110.0 | 11.7 | 126.3 | 6.8 | 126.2 | 0.5 | 0.6 | 150.2 | 43.7 | 20.7 | 6.2 | 0.2 | 0.1 | 0.4 |
| 59 |  | 0.97 | 8.10 | 73.4 | 5.7 | 91.6 | 3.7 | 88.3 | 0.4 | 0.4 | 100.7 | 31.2 | 14.5 | 4.7 | 0.0 | 0.0 | 0.2 |
| 63 | 63 | 1.43 | 8.02 | 113.0 | 11.5 | 140.7 | 4.8 | 122.6 | 0.4 | 0.9 | 145.6 | 43.1 | 7.3 | 7.0 | 0.0 | 0.0 | 0.2 |
| 66 |  | 1.11 | 8.09 | 90.4 | 7.1 | 114.4 | 3.3 | 100.9 | 0.4 | 0.6 | 115.6 | 36.0 | 6.3 | 5.7 | 0.0 | 0.0 | 0.1 |
| 69 |  | 0.50 | 8.17 | 40.6 | 1.4 | 51.8 | 0.9 | 45.1 | 0.2 | 0.2 | 51.9 | 16.7 | 3.2 | 2.9 | 0.0 | 0.0 | 0.1 |
|  | 70(11/10) | 1.48 |  | 108.0 | 22.6 | 109.8 | 15.5 | 122.3 | 0.7 | 1.5 | 123.5 | 33.3 | 15.4 | 5.9 | 0.2 | 0.4 | 2.44 |
| 72 |  | 1.45 | 7.98 | 118.4 | 7.8 | 142.2 | 3.0 | 129.3 | 0.4 | 0.8 | 146.5 | 47.3 | 9.0 | 8.1 | 0.0 | 0.0 | 0.3 |
| 76 |  | 1.12 | 8.20 | 92.6 | 2.7 | 109.8 | 1.5 | 100.4 | 0.3 | 0.5 | 109.1 | 37.9 | 7.8 | 7.0 | 0.0 | 0.0 | 0.2 |
| 79 | 79 | 1.41 | 7.89 | 94.6 | 0.0 | 129.0 | 2.8 | 122.4 | 0.4 | 0.8 | 132.0 | 46.3 | 9.1 | 8.5 | 0.0 | 0.0 | 0.3 |
| 83 |  | 0.74 | 8.01 | 61.4 | 1.2 | 75.1 | 0.7 | 65.9 | 0.2 | 0.4 | 65.2 | 25.6 | 5.9 | 5.1 | 0.0 | 0.0 | 0.2 |
|  | 84(11/24) | 1.45 |  | 106.7 | 22.1 | 107.6 | 15.2 | 119.8 | 0.6 | 1.4 | 121.2 | 32.6 | 15.1 | 5.8 | 0.2 | 0.4 | 2.39 |
| 86 |  | 1.44 | 7.95 | 120.9 | 6.9 | 134.9 | 2.0 | 129.2 | 0.3 | 0.9 | 134.8 | 49.2 | 10.0 | 9.3 | 0.0 | 0.0 | 0.3 |
| 90 |  | 0.74 | 8.08 | 63.5 | 0.6 | 69.1 | 0.2 | 68.0 | 0.2 | 0.5 | 63.7 | 27.5 | 6.2 | 5.6 | 0.0 | 0.0 | 0.1 |
|  | 91(12/1) | 1.5 |  | 110.3 | 22.9 | 111.3 | 15.7 | 124.0 | 0.7 | 1.5 | 125.3 | 33.7 | 15.6 | 6.0 | 0.2 | 0.4 | 2.47 |
| 93 |  | 1.46 | 8.05 | 126.6 | 3.9 | 131.2 | 0.7 | 131.3 | 0.3 | 1.0 | 122.6 | 53.7 | 12.2 | 11.1 | 0.0 | 0.0 | 0.2 |
| 97 | 97 | 1.43 | 7.72 | 141.7 | 15.0 | 147.2 | 2.7 | 142.5 | 0.2 | 1.2 | 158.4 | 58.1 | 10.8 | 10.5 | 0.0 | 0.1 | 0.3 |
| 100 |  | 1.02 | 8.01 | 90.1 | 2.6 | 85.3 | 0.6 | 89.8 | 0.2 | 0.7 | 84.9 | 36.9 | 8.2 | 7.8 | 0.0 | 0.0 | 0.2 |
| 104 | 104 | 1.49 | 7.80 | 132.1 | 7.7 | 113.2 | 2.5 | 132.9 | 0.2 | 1.0 | 116.2 | 51.0 | 11.5 | 11.1 | 0.0 | 0.1 | 0.5 |
| 107 |  | 1.00 | 8.31 | 90.5 | 1.1 | 74.6 | 0.5 | 88.3 | 0.2 | 0.6 | 72.3 | 35.3 | 8.6 | 7.9 | 0.0 | 0.0 | 0.3 |
| 111 |  | 0.35 | 7.76 | 31.3 | 1.7 | 26.1 | 0.5 | 31.4 | 0.1 | 0.2 | 24.8 | 12.1 | 2.8 | 2.7 | 0.0 | 0.0 | 0.1 |
