# Supplementary material for: Characterizing nutrient uptake kinetics for efficient crop production during Solanum lycopersicum var. cerasiforme Alef. growth in a closed indoor hydroponic system
Source: PLoS One. 2017 May 9;12(5):e0177041. doi: 10.1371/journal.pone.0177041 (PMC5423622; doi:10.1371/journal.pone.0177041)
Supplement: S6 Table — (DOCX) [file pone.0177041.s008.docx]

S6 Table. Comparison of ion concentrations determined by analytical instruments [i.e., cations by inductively coupled plasma-optical emission spectroscopy (ICP-OES), anions by ion chromatography (IC)] and on-site measurements [i.e., K^+^, Na^+^, Cl^–^ by ion-specific electrodes (ISE) and NO_3_^–^ and PO_4_^3–^ by commercial kit]

|  | K^+^ (ppm) | | Na^+^ (ppm) | | Cl^+^ (ppm) | | NO_3_^-^, T-N (ppm) | | PO_4_^3-^, T-P (ppm) | |
| --- | --- | --- | --- | --- | --- | --- | --- | --- | --- | --- |
| Time | ISE | ICP | ISE | ICP | ISE | ICP | KIT | ICP | KIT | ICP |
| 0 | 155.0 | 101.1 | 7.5 | 12.6 | 19.2 | 12.7 | 126.0 | 89.0 | 20.1 | 18.5 |
| 1 | 154.1 | 96.4 | 6.7 | 12.4 | 15.6 | 11.9 | 102.9 | 87.5 | 21.1 | 11.7 |
| 2 | 142.9 | 93.9 | 6.2 | 12.0 | 15.7 | 12.0 | 95.7 | 83.5 | 17.9 | 16.4 |
| 4 | 134.2 | 90.8 | 5.9 | 11.4 | 15.5 | 11.5 | 101.3 | 80.7 | 15.8 | 14.6 |
| 6 | 131.8 | 91.4 | 5.9 | 11.1 | 16.1 | 11.6 | 91.9 | 81.5 | 16.5 | 17.2 |
| 8 | 116.9 | 81.1 | 5.2 | 10.1 | 14.8 | 10.2 | 91.5 | 74.5 | 14.1 | 13.3 |
| 10 | 89.3 | 78.0 | 6.1 | 10.0 | 14.1 | 10.1 | 81.0 | 73.3 | 10.2 | 11.8 |
| 12 | 125.0 | 102.7 | 8.9 | 13.5 | 19.2 | 13.8 |  | 98.1 |  | 14.0 |
| 14 | 118.9 | 92.0 | 9.0 | 12.8 | 17.9 | 13.0 | 112.1 | 95.2 | 9.3 | 13.3 |
| 16 | 130.1 | 113.7 | 12.6 | 12.5 | 18.6 | 12.2 | 138.5 | 109.2 | 14.7 | 19.6 |
| 18 | 134.1 | 118.8 | 16.3 | 12.9 | 18.2 | 12.9 | 138.8 | 113.0 | 14.7 | 20.7 |
| 20 | 128.7 | 117.2 | 13.8 | 13.1 | 17.5 | 12.4 | 115.3 | 112.5 | 14.6 | 22.6 |
| 22 | 120.0 | 104.1 | 11.1 | 11.7 | 16.1 | 11.3 | 95.0 | 100.4 | 12.6 | 17.7 |
| 24 | 101.3 | 90.0 | 8.4 | 11.2 | 14.2 | 9.9 | 99.9 | 88.1 | 10.6 | 13.7 |
| 27 | 17.0 | 54.7 | 1.5 | 7.0 | 8.5 | 6.3 | 52.9 | 53.8 | 5.9 | 7.8 |
| 33 | 10.8 | 44.5 | 1.4 | 4.2 | 5.7 | 5.5 | 34.2 | 34.7 | 4.8 | 3.8 |
| 36 | 25.1 | 103.6 | 2.2 | 9.7 | 10.1 | 9.4 | 51.9 | 83.5 | 7.2 | 8.3 |
| 39 | 15.5 | 62.1 | 1.3 | 5.5 | 6.0 | 5.5 | 61.4 | 51.3 | 3.3 | 3.4 |
| 42 | 11.2 | 50.6 | 1.2 | 5.1 | 4.1 | 3.6 | 44.5 | 38.5 | 2.3 | 3.1 |
| 45 | 27.6 | 122.6 | 3.2 | 14.1 | 13.5 | 11.3 | 96.8 | 86.6 | 2.5 | 6.5 |
| 49 | 21.5 | 96.2 | 2.6 | 11.3 | 7.0 | 5.9 | 78.6 | 73.4 | 3.6 | 5.6 |
| 52 | 14.8 | 67.3 | 1.9 | 9.8 | 4.4 | 3.5 | 55.7 | 52.3 | 3.2 | 2.8 |
| 56 | 33.1 | 150.2 | 4.3 | 20.7 | 8.8 | 6.8 | 124.5 | 110.0 | 5.8 | 11.7 |
| 59 | 21.9 | 100.7 | 3.1 | 14.5 | 4.9 | 3.7 | 82.1 | 73.4 | 3.2 | 5.7 |
| 63 | 37.6 | 145.6 | 8.8 | 7.3 | 7.1 | 4.8 | 114.0 | 113.0 | 5.4 | 11.5 |
| 66 | 29.2 | 115.6 | 5.9 | 6.3 | 5.3 | 3.3 | 94.2 | 90.4 | 3.2 | 7.1 |
| 69 | 12.3 | 51.9 | 2.7 | 3.2 | 2.2 | 0.9 | 41.1 | 40.6 | 1.0 | 1.4 |
| 72 | 35.9 | 146.5 | 8.3 | 9.0 | 5.0 | 3.0 | 118.8 | 118.4 | 2.9 | 7.8 |
| 76 | 25.8 | 109.1 | 7.6 | 7.8 | 3.0 | 1.5 | 91.3 | 92.6 | 4.3 | 2.7 |
| 79 | 30.8 | 132.0 | 9.4 | 9.1 | 5.3 | 2.8 | 115.3 | 94.6 | 5.1 | - |
| 83 | 16.2 | 65.2 | 5.7 | 5.9 | 1.8 | 0.7 | 61.5 | 61.4 | 2.2 | 1.2 |
| 86 | 29.4 | 134.8 | 10.2 | 10.0 | 4.2 | 2.0 | 115.8 | 120.9 | 4.9 | 6.9 |
| 90 | 14.0 | 63.7 | 6.1 | 6.2 | 1.7 | 0.2 | 52.1 | 63.5 | 4.0 | 0.6 |
| 93 | 26.4 | 122.6 | 13.7 | 12.2 | 1.8 | 0.7 | 113.5 | 126.6 | 9.2 | 3.9 |
| 97 | 62.0 | 158.4 |  | 10.8 | 7.1 | 2.7 | 118.0 | 141.7 | 6.9 | 15.0 |
| 100 | 18.9 | 84.9 | 9.2 | 8.2 | 1.6 | 0.6 | 84.1 | 90.1 | 6.0 | 2.6 |
| 104 | 26.2 | 116.2 | 12.4 | 11.5 | 4.3 | 2.5 | 124.8 | 132.1 | 5.6 | 7.7 |
| 107 | 16.7 | 72.3 | 9.0 | 8.6 | 1.2 | 0.5 | 76.9 | 90.5 | 4.7 | 1.1 |
| 111 | 5.9 | 24.8 | 3.0 | 2.8 | 1.1 | 0.5 | 27.5 | 31.3 | 1.9 | 1.7 |
