# Supplementary material for: Characterizing nutrient uptake kinetics for efficient crop production during Solanum lycopersicum var. cerasiforme Alef. growth in a closed indoor hydroponic system
Source: PLoS One. 2017 May 9;12(5):e0177041. doi: 10.1371/journal.pone.0177041 (PMC5423622; doi:10.1371/journal.pone.0177041)
Supplement: S7 Table — (DOCX) [file pone.0177041.s009.docx]

S7 Table. Variation in the saturation index (SI) of several carbonate and phosphate minerals during tomato growth in the closed hydroponic system.

| **Day** | **pH** | **Octacalcium  phosphate** | **Hydroxylapatite** | **Calcite** | **Dolomite** | **Calcium phosphate hydroxide** |
| --- | --- | --- | --- | --- | --- | --- |
|  |  | **Ca_4_H(PO_4_)_3_⋅3H_2_O** | **Ca_5_(PO_4_)_3_(OH)** | **CaCO_3_** | **CaMg(CO_3_)_2_** | **Ca_3_(PO_4_)_2_** |
| 0 | 7.58 | -1.594 | 7.049 | 1.723 | 3.449 | 0.267 |
| 1 | 7.69 | 1.216 | 11.222 | -0.414 | -1.027 | 2.595 |
| 2 | 7.74 | 2.058 | 12.032 | -0.446 | -1.115 | 3.146 |
| 4 | 7.60 | 1.851 | 11.665 | -0.606 | -1.417 | 2.954 |
| 6 | 7.68 | 1.895 | 11.755 | -0.560 | -1.323 | 2.999 |
| 8 | 7.65 | 0.921 | 10.751 | -0.590 | -1.388 | 2.340 |
| 10 | 7.66 | 0.884 | 10.76 | -0.544 | -1.249 | 2.330 |
| 12 | 7.48 | 0.501 | 9.939 | -0.982 | -2.144 | 1.929 |
| 14 | 6.79 | -1.637 | 6.431 | -2.352 | -4.844 | 0.047 |
| 16 | 6.69 | -0.865 | 7.064 | -2.491 | -5.176 | 0.515 |
| 18 | 5.58 | -5.661 | 0.080 | -4.679 | -9.549 | -3.411 |
| 20 | 5.80 | -4.491 | 1.752 | -4.177 | -8.561 | -2.464 |
| 22 | 5.98 | -3.727 | 2.854 | -3.840 | -7.893 | -1.842 |
| 24 | 6.71 | -1.959 | 5.908 | -2.553 | -5.308 | -0.235 |
| 27 | 7.05 | -1.438 | 6.881 | -2.101 | -0.586 | -2.281 |
| 33 | 7.67 | -42.015 | -32.528 | -0.933 | -2.077 | -26.399 |
| 36 | 7.42 | 0.445 | 9.911 | -0.954 | -2.068 | 1.901 |
| 39 | 7.75 | -41.389 | -31.546 | -0.577 | -1.315 | -25.863 |
| 42 | 7.87 | -41.248 | -31.216 | -0.609 | -0.899 | -25.706 |
| 45 | 7.75 | 1.255 | 11.306 | -0.369 | -0.852 | 2.636 |
| 49 | 7.92 | 1.330 | 11.597 | -0.153 | -0.424 | 2.758 |
| 52 | 8.07 | -40.561 | -29.988 | 0.154 | 0.206 | -25.067 |
| 56 | 7.93 | 1.794 | 12.342 | 0.128 | 0.165 | 3.161 |
| 59 | 8.22 | 2.006 | 12.971 | 0.544 | 1.007 | 3.441 |
| 63 | 8.08 | 2.090 | 12.969 | 0.459 | 0.831 | 3.469 |
| 66 | 8.04 | 1.709 | 12.284 | 0.154 | 0.232 | 3.113 |
| 69 | 8.20 | -40.631 | -29.938 | 0.273 | 0.491 | -25.074 |
| 72 | 8.00 | 1.974 | 12.717 | 0.322 | 0.565 | 3.346 |
| 76 | 8.20 | -39.894 | -28.868 | 0.606 | 1.165 | -24.471 |
| 79 | 7.91 | -40.033 | -29.29 | 0.323 | 0.58 | -24.659 |
| 83 | 8.07 | -40.473 | -29.852 | 0.201 | 0.342 | -24.993 |
| 86 | 8.01 | 1.967 | 12.714 | 0.327 | 0.597 | 3.342 |
| 90 | 8.11 | -40.406 | -29.752 | 0.234 | 0.422 | -24.937 |
| 93 | 8.13 | -39.882 | -28.961 | 0.501 | 0.973 | -24.499 |
| 97 | 7.68 | 2.207 | 12.271 | -0.356 | -0.745 | 3.275 |
| 100 | 8.03 | -40.405 | -29.866 | 0.119 | 0.216 | -24.975 |
| 104 | 7.86 | 1.652 | 12.051 | -0.021 | -0.094 | 3.017 |
| 107 | 8.36 | -39.798 | -28.597 | 0.781 | 1.512 | -24.35 |
| 111 | 7.76 | -42.022 | -32.464 | -0.862 | -1.792 | -26.38 |
