# Supplementary material for: Characterizing nutrient uptake kinetics for efficient crop production during Solanum lycopersicum var. cerasiforme Alef. growth in a closed indoor hydroponic system
Source: PLoS One. 2017 May 9;12(5):e0177041. doi: 10.1371/journal.pone.0177041 (PMC5423622; doi:10.1371/journal.pone.0177041)
Supplement: S8 Table — (DOCX) [file pone.0177041.s010.docx]

S8 Table. Ca/P ratio, morphology, and mineralogy of solid particles precipitated from nutrient solution.

| pH | Ca/P ratio (liquid) | Ca/P ratio (powder) | Morphology (SEM) | Mineralogy  (XRD) |
| --- | --- | --- | --- | --- |
| 8.0–8.5 | 1.21 | 1.24 | Plates  Amorphous clusters | Not detected |
