# Supplementary material for: Characterizing nutrient uptake kinetics for efficient crop production during Solanum lycopersicum var. cerasiforme Alef. growth in a closed indoor hydroponic system
Source: PLoS One. 2017 May 9;12(5):e0177041. doi: 10.1371/journal.pone.0177041 (PMC5423622; doi:10.1371/journal.pone.0177041)
Supplement: S9 Table — (DOCX) [file pone.0177041.s011.docx]

S9 Table. Pearson correlation coefficient determination between electrical conductivity, pH and major and minor cations and anions concentration during the tomato plant growth

| Para-  meter | EC | pH | NO_3_^-^ | PO_4_^3-^ | SO_4_^2-^ | Cl^-^ | Ca^2+^ | Cu^2+^ | Fe | K^+^ | Mg^2+^ | Na^+^ | Si | Zn^2+^ | Mn | NH_4_ |
| --- | --- | --- | --- | --- | --- | --- | --- | --- | --- | --- | --- | --- | --- | --- | --- | --- |
|  | mS cm^-1^ | - | mgN/L | mgP/L | mg/L | mg/L | mg/L | mg/L | mg/L | mg/L | mg/L | mg/L | mg/L | mg/L | mg/L | mgN/L |
| EC | 1.00 | - | - | - | - | - | - | - | - | - | - | - | - | - | - | - |
| pH | -0.21 | 1.00 | - | - | - | - | - | - | - | - | - | - | - | - | - | - |
| NO_3_^-^ | 0.95 | -0.22 | 1.00 | - | - | - | - | - | - | - | - | - | - | - | - | - |
| PO_4_^3-^ | 0.50 | -0.73 | 0.47 | 1.00 | - | - | - | - | - | - | - | - | - | - | - | - |
| SO_4_^2-^ | 0.89 | -0.27 | 0.91 | 0.43 | 1.00 | - | - | - | - | - | - | - | - | - | - | - |
| Cl^-^ | 0.29 | -0.66 | 0.15 | 0.84 | 0.12 | 1.00 | - | - | - | - | - | - | - | - | - | - |
| Ca^2+^ | 0.97 | -0.09 | 0.96 | 0.38 | 0.91 | 0.10 | 1.00 | - | - | - | - | - | - | - | - | - |
| Cu^2+^ | 0.21 | -0.37 | 0.18 | 0.05 | 0.41 | 0.02 | 0.22 | 1.00 | - | - | - | - | - | - | - | - |
| Fe | 0.76 | -0.01 | 0.80 | 0.40 | 0.67 | 0.10 | 0.80 | -0.20 | 1.00 | - | - | - | - | - | - | - |
| K^+^ | 0.93 | -0.09 | 0.90 | 0.40 | 0.94 | 0.15 | 0.95 | 0.33 | 0.70 | 1.00 | - | - | - | - | - | - |
| Mg^2+^ | 0.82 | 0.19 | 0.87 | 0.03 | 0.83 | -0.28 | 0.92 | 0.18 | 0.72 | 0.86 | 1.00 | - | - | - | - | - |
| Na^+^ | 0.70 | -0.32 | 0.60 | 0.59 | 0.52 | 0.56 | 0.62 | 0.21 | 0.40 | 0.63 | 0.40 | 1.00 | - | - | - | - |
| Si | 0.55 | 0.57 | 0.61 | -0.27 | 0.47 | -0.52 | 0.68 | -0.20 | 0.64 | 0.56 | 0.87 | 0.13 | 1.00 | - | - | - |
| Zn^2+^ | 0.16 | -0.73 | 0.05 | 0.64 | 0.10 | 0.79 | 0.01 | 0.43 | -0.16 | 0.08 | -0.29 | 0.56 | -0.61 | 1.00 | - | - |
| Mn | 0.33 | -0.42 | 0.26 | 0.77 | 0.11 | 0.72 | 0.23 | -0.37 | 0.48 | 0.17 | -0.07 | 0.49 | -0.11 | 0.45 | 1.00 | - |
| NH_4_ | 0.26 | -0.14 | 0.14 | 0.60 | -0.04 | 0.68 | 0.13 | -0.60 | 0.37 | 0.06 | -0.13 | 0.40 | -0.04 | 0.26 | 0.86 | 1.00 |
| Note: Strength of the positive and negative correlation was evaluated using the Evans (1996) guidelines: a) very weak: 0.00-0.019, b) weak: 0.20-0.39, c) moderate: 0.40-0.59, d) strong: 0.60-0.79, e) very strong: 0.80-1.0.  Evans, J. D. (1996). Straightforward statistics for the behavioral sciences. Pacific Grove, CA: Brooks/Cole  Publishing. | | | | | | | | | | | | | | | | |
|  |  |  |  |  |  |  |  |  |  |  |  |  |  |  |  |  |
