# Supplementary material for: Characterizing nutrient uptake kinetics for efficient crop production during Solanum lycopersicum var. cerasiforme Alef. growth in a closed indoor hydroponic system
Source: PLoS One. 2017 May 9;12(5):e0177041. doi: 10.1371/journal.pone.0177041 (PMC5423622; doi:10.1371/journal.pone.0177041)
Supplement: S10 Table — (DOCX) [file pone.0177041.s012.docx]

S10 Table. Two-sample t-test (unequal variances) for p-value determination (significance level of 5% or α of 0.05) between major nutrients uptake rate (mg L^–1^ d^–1^) at transplanting phase of plants growth

| Ions | NO_3_^-^ | PO_4_^3-^ | SO_4_^2-^ | K^+^ | Ca^2+^ | Mg^2+^ |
| --- | --- | --- | --- | --- | --- | --- |
| NO_3_^-^ | - | 0.183 | 0.909 | 0.853 | 0.922 | 0.226 |
| PO_4_^3-^ | 0.183 | - | 0.291 | 0.190 | 0.109 | 0.338 |
| SO_4_^2-^ | 0.909 | 0.291 | - | 0.786 | 0.964 | 0.355 |
| K^+^ | 0.853 | 0.190 | 0.786 | - | 0.770 | 0.227 |
| Ca^2+^ | 0.922 | 0.109 | 0.964 | 0.770 | - | 0.135 |
| Mg^2+^ | 0.226 | 0.338 | 0.355 | 0.227 | 0.135 | - |
