# Supplementary material for: Characterizing nutrient uptake kinetics for efficient crop production during Solanum lycopersicum var. cerasiforme Alef. growth in a closed indoor hydroponic system
Source: PLoS One. 2017 May 9;12(5):e0177041. doi: 10.1371/journal.pone.0177041 (PMC5423622; doi:10.1371/journal.pone.0177041)
Supplement: S11 Table — (DOCX) [file pone.0177041.s013.docx]

S11 Table. Two-sample t-test (unequal variances) for p-value determination (significance level of 5% or α of 0.05) between major nutrients uptake rate (mg L^–1^ d^–1^) at flowering and fruit-set phases of tomato growth

| Ions | NO_3_^-^ | PO_4_^3-^ | SO_4_^2-^ | K^+^ | Ca^2+^ | Mg^2+^ |
| --- | --- | --- | --- | --- | --- | --- |
| NO_3_^-^ | - | 0.018 | 0.412 | 0.225 | 0.449 | 0.054 |
| PO_4_^3-^ | 0.018 | - | 0.027 | 0.021 | 0.011 | 0.037 |
| SO_4_^2-^ | 0.412 | 0.027 | - | 0.669 | 0.808 | 0.055 |
| K^+^ | 0.225 | 0.021 | 0.669 | - | 0.478 | 0.038 |
| Ca^2+^ | 0.449 | 0.011 | 0.808 | 0.478 | - | 0.022 |
| Mg^2+^ | 0.054 | 0.037 | 0.055 | 0.038 | 0.022 | - |
