# Supplementary material for: Characterizing nutrient uptake kinetics for efficient crop production during Solanum lycopersicum var. cerasiforme Alef. growth in a closed indoor hydroponic system
Source: PLoS One. 2017 May 9;12(5):e0177041. doi: 10.1371/journal.pone.0177041 (PMC5423622; doi:10.1371/journal.pone.0177041)
Supplement: S12 Table — (DOCX) [file pone.0177041.s014.docx]

S12 Table. Two-sample t-test (unequal variances) for p-value (significance level of 5% or α of 0.05) and linear regression coefficient determination between ion concentrations determined by various analytical instruments [i.e., cations by inductively coupled plasma-optical emission spectroscopy (ICP-OES), anions by ion chromatography (IC)] and on-site measurements [i.e., K^+^, Na^+^, Cl^–^ by ion-specific electrodes (ISE) and NO_3_^–^ and PO_4_^3–^ by commercial kit]

| Ions | Analysis Method | p-value | Regression coefficient (R^2^) |
| --- | --- | --- | --- |
| K^+^ | ISE and ICP | 0.001 | 0.042 |
| Na^+^ | ISE and ICP | 0.000 | 0.139 |
| Cl^-^ | ISE and ICP | 0.035 | 0.968 |
| NO_3_^-^ | KIT and ICP | 0.532 | 0.787 |
| PO_4_^3-^ | KIT and ICP | 0.398 | 0.652 |
